# Targeting CDC42 Protects Mitochondrial Function through KLF2/HIF-1α/PINK1 Signaling in Acute Kidney Injury

**DOI:** 10.7150/ijbs.125930

**Published:** 2026-01-01

**Authors:** Xue Zhou, Xian Fu, Yi-Wen Meng, Ping Dai, Qing Jiang, Hou-Hua Yin, Qing-Jin Pan, Ai-Zhi Lin, Kai-Di Ni, Zi-Guo Luo, Ru-Yu Liang, Yi-Yu Chen, Hai-Xin Yuan, Jun-Yan Liu

**Affiliations:** 1 Medical Examination Centre of the First Affiliated Hospital and CNTTI of College of Pharmacy, Chongqing Medical University, Chongqing 400016, China.; 2 Basic Medicine Research and Innovation Center for Novel Target and Therapeutic Intervention (CNTTI), Ministry of Education, Chongqing 400016, China.; 3 Department of Chemical Biology, College of Pharmacy, Chongqing Medical University, Chongqing 400016, China.; 4 Sci-Tech Innovation Center, Chongqing Medical University, Chongqing 400016, China.; 5 Department of Anesthesia of the Second Affiliated Hospital, Chongqing Medical University, Chongqing, 400061, China.

**Keywords:** acute kidney injury, cell division cycle 42, mitochondrial dysfunction, kruppel-like factor 2, hypoxia-inducible factor-1alpha, oxidative stress

## Abstract

Acute kidney injury (AKI) is a severe clinical syndrome strongly associated with mitochondrial dysfunction and oxidative stress, yet effective therapies remain elusive. Here, we identify cell division cycle 42 (CDC42) as a critical mediator of AKI. Analysis of human single-cell RNA sequencing (scRNA-seq) dataset revealed marked upregulation of CDC42 in renal tubular epithelial cells (RTECs), which was validated in murine models of cisplatin- and ischemia-reperfusion-induced AKI. Pharmacological inhibition, conditional knockdown, or genetic ablation of CDC42 significantly alleviated renal injury, preserved mitochondrial function, and reduced reactive oxygen species (ROS) both *in vivo* and *in vitro*. Mechanistically, transcriptomic analysis, bioinformatic analysis, dual-luciferase reporter assays, ChIP assays and cellular functional validation revealed that CDC42 suppression activated a *KLF2*/*HIF-1α*/*PINK1* transcriptional cascade, thereby promoting mitophagy and restoring mitochondrial homeostasis. Functional assays supported that this pathway plays a pivotal role in protecting RTECs from oxidative damage. Collectively, these findings uncover a previously unrecognized role of CDC42 in AKI pathogenesis and highlight CDC42 inhibition as a promising therapeutic strategy for mitigating mitochondrial damage and improving renal outcomes.

## Introduction

Acute kidney injury (AKI), characterized by rapid deterioration of renal dysfunction, poses a critical healthcare challenge due to its high morbidity, persistently elevated mortality over decades, and substantial socioeconomic burden 1-3. Affecting 10-15% of hospitalized patients and over 50% of ICU admissions, AKI is strongly associated with adverse short-term outcomes, including a 5.6-fold higher 28-day mortality 4, and long-term risks of chronic kidney disease and mortality 5. Current management is largely limited to supportive care, highlighting the urgent need to explore novel prophylactic and therapeutic strategies.

Mitochondrial dysfunction and oxidative stress are central drivers of AKI pathogenesis, particularly in renal tubular epithelial cells (RTECs), which are highly metabolically active and rely on mitochondrial integrity to sustain reabsorption and energy production 6-9. Under AKI-related stress, mitochondria become dysfunctional and generate excessive levels of reactive oxygen species (ROS), which are estimated to contribute up to 90% of cellular ROS production 6,7,10. The resulting cellular ROS surge exacerbates mitochondrial damage and RTECs injury 1,8, creating a self-perpetuating cycle of oxidative damage and organ dysfunction. Therefore, strategy break this vicious cycle and restore mitochondrial homeostasis represent promising therapeutic avenues. However, the molecular mechanisms regulating redox-sensitive mitochondrial maintenance in RTECs remain incompletely understood.

In our ongoing effort to explore the new therapeutic strategies for kidney diseases 11-13, integrated analysis of published human single-cell RNA sequencing (scRNA-seq) data highlighted a potential role of cell division cycle 42 (CDC42). CDC42 is a key regulator of actin cytoskeleton dynamics, controlling essential cellular processes such as shape, migration, cell cycle progression and vesicle trafficking 14-16. Moreover, CDC42 functions as a cellular signaling switch, modulating multiple signaling pathways and governing diverse cellular functions 17. Its broad biological functions make it essential for renal development 18 and an attractive therapeutic target in several malignancies, including gastric 19, colorectal 20, and breast cancers 21,22. While CDC42 has been reported to be involved in mitochondrial fission 23, its role in mitochondrial homeostasis, particularly in redox balance during AKI, remains unknown.

Here, we report for the first time that CDC42 inhibition activates KLF2/HIF-1α/PINK1 signaling axis to promote mitophagy, restore mitochondrial function, and attenuate AKI by reducing oxidative stress. Using murine models of cisplatin- and ischemic-reperfusion (I/R)-induced AKI, as well as cisplatin-caused injury in human RTECs, we show that pharmacological and genetic suppression of CDC42 significantly attenuates mitochondrial dysfunction and renal injury. Collectively, these findings identify CDC42 as a previously unrecognized regulator of redox-sensitive mitochondrial homeostasis and offers mechanistic insights into its therapeutic potential in AKI.

## Methods

### Study approval

All animal experiments in this study were approved by the Animal Research and Ethics Committee of Chongqing Medical University (IACUC-CQMU-2023-11016) following the National Institutes of Health Guide for the Care and Use of Laboratory Animals.

### Animals

Male mice (C57BL/6J, 8-weeks old) were purchased from GemPharmatech Co., Ltd. (Chengdu, China). *Cdc42*^flox/-^ and Cdh16-Cre mice, both with a C57BL/6 background, were purchased from the Shanghai Model Organisms Center, Inc. (Shanghai, China). *Cdc42*^flox/-^ mice were crossed with Cdh16-Cre mice using the Cre-loxP system to generate renal tubular epithelial-specific *Cdc42* knockdown mice *Cdc42^+/-^*; Cdh16-Cre (*Cdc42*^Cdh16 KD^), and *Cdc42*^flox/flox^ mice (*Cdc42*^f/f^) were generated by mating *Cdc42*^flox/-^ mice with* Cdc42*^flox/-^ mice. The genotypes of mice were identified by PCR analysis of the DNA of tail tissues with the primer sequences presented in Supplementary Table 1.

**Figure 1:**
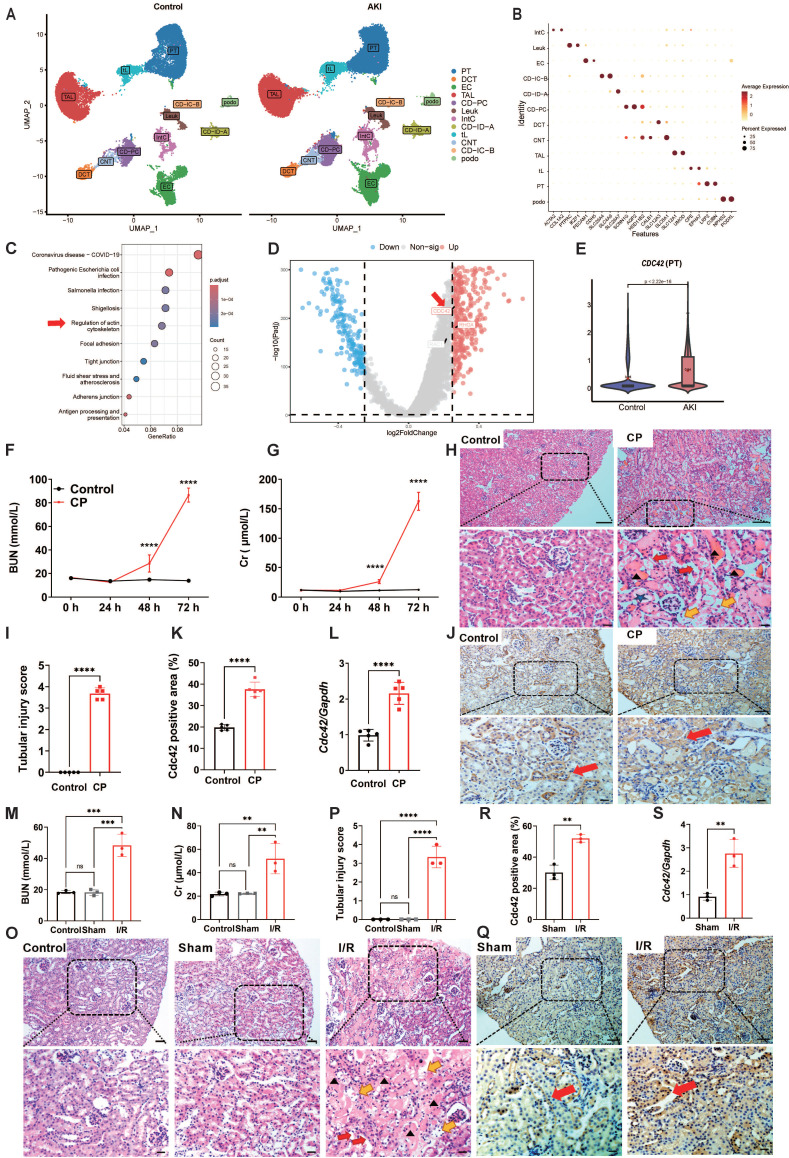
** CDC42 was upregulated in the kidneys of AKI patients, CP-AKI mice and I/R-AKI mice. (A)** Uniform manifold approximation and projection (UMAP) of 96527 cells from individuals with controls (left) and AKI (right). Data were originated from the scRNA-seq dataset reported by Christian *et al.* 24. (Podo, podocytes; PT, proximal tubule; tL, thin limb; TAL, thick ascending limb; DCT, distal convoluted tubule; CNT, connecting tubule; CD-PC/IC-A/IC-B, collecting duct principal/intercalated cells type A and B; Leuk, leukocytes; IntC, interstitial cells); **(B)** Dot plot showing the representative maker genes of each cell cluster; **(C)** KEGG pathway enrichment analysis of PT cells from control and AKI group; **(D)** Volcano plot of PT cells in the control and AKI group; **(E)** Violin plot indicated significantly upregulation of *CDC42* in PT cells from AKI group; **(F)** BUN and blood Cr **(G)** concentrations in mice were increased upon CP-challenge time-dependently (n = 5, means ± SEM); **(H)** Representative H&E-stained kidney sections from CP-treated mice showed tubular injury features such as cast formation (black arrows), brush border loss (red arrows), epithelial cell necrosis (yellow arrows), and tubular dilation (blue pentagram). n = 5, scale bars: 100 μm (overview), 20 μm (magnified); (**I**) Renal tubular injury scores of **(H)**;** (J-K)** Immunohistochemical staining revealed elevated Cdc42 expression (brown signal, red arrows) in renal tubules of CP-AKI mice (n = 5). Scale bars: 50 μm (overview), 20 μm (magnified); **(L)** Renal *Cdc42* mRNA levels were significantly increased in CP-induced AKI mice (n = 5);** (M)** BUN and Cr **(N)** concentrations were increased in I/R-induced AKI mice (n = 3); **(O)** H&E staining demonstrated severe kidney injury in I/R mice (n = 3). Scale bars: 50 μm (overview), 20 μm (magnified); **(P)** Renal tubular injury scores of **(O)**;** (Q-R)** IHC staining implied increased Cdc42 expression in I/R-AKI mice (n = 3). Scale bars: 50 μm (overview), 20 μm (magnified); **(S)** Renal *Cdc42* mRNA levels were increased in I/R-AKI mice (n = 3); Data were presented as means ± SD unless other noted. * 0.01 <* p* ≤ 0.05, ** 0.001< *p* ≤ 0.01, *** 0.0001 <*p* ≤ 0.001, **** *p* ≤ 0.0001.

**Figure 2:**
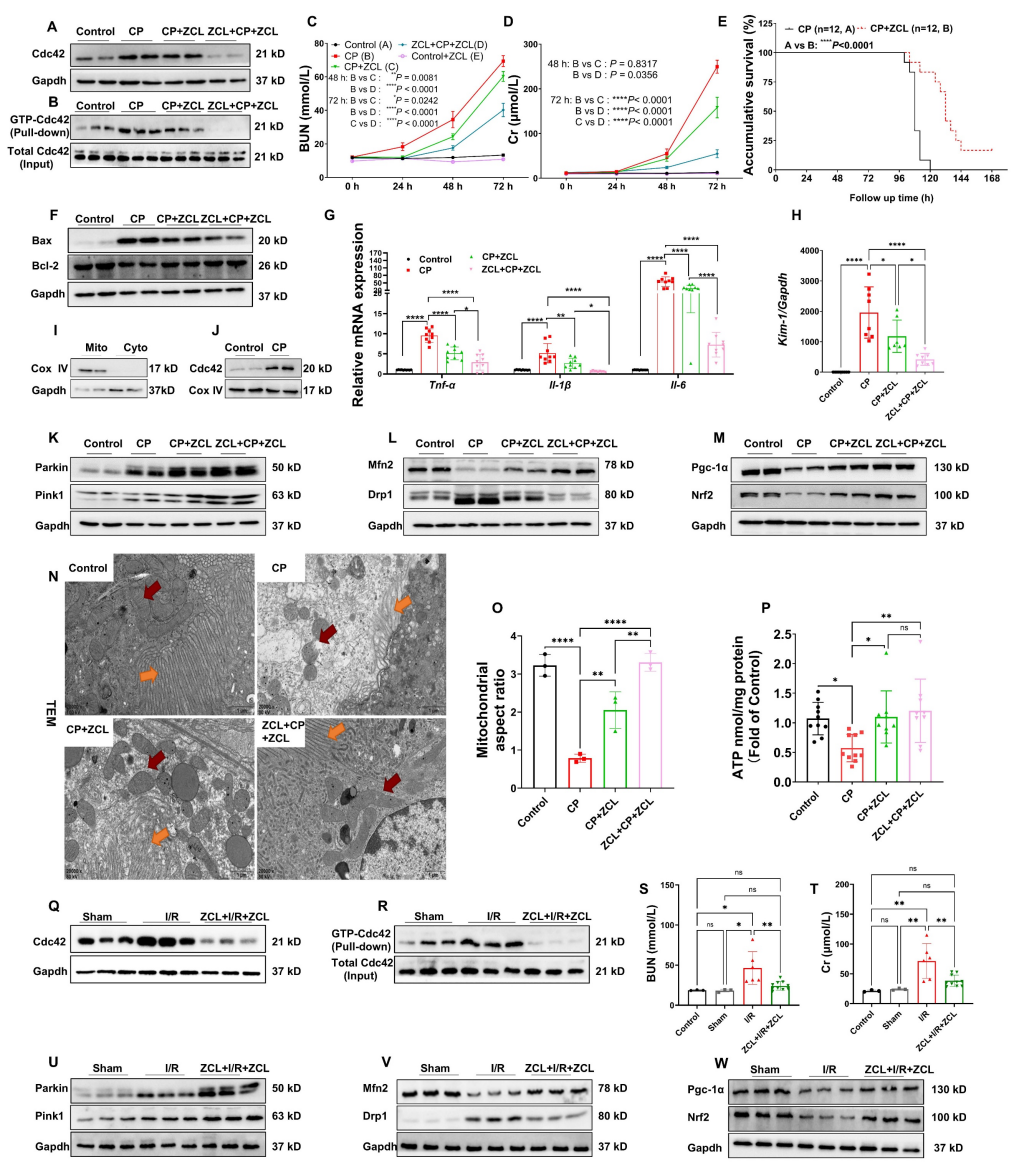
** Cdc42 inhibition by ZCL278 attenuated renal injury and mitochondrial dysfunction in AKI mice. (A-B)** ZCL278 treatment inhibited the elevated renal Cdc42 protein expression **(A)** and GTP-Cdc42 levels **(B)** in CP-induced AKI mice (n  =  6);** (C-D)** ZCL278 attenuated CP-induced increase in BUN** (C)** and Cr levels** (D)** (n = 5 in control+ZCL278 group, n = 7~9 in other groups, means ± SEM); **(E)** ZCL278 improved survival in CP-challenged mice;** (F)** ZCL278 reversed CP-induced changes in the renal protein expression of apoptotic indicator Bax and the antiapoptotic indicator Bcl-2 (n = 6); **(G, H)** ZCL278 reversed CP-induced increases in renal mRNA levels of pro-inflammatory *Il-1β*, *Il-6* and *Tnf-α* **(G)** and *Kim-1* **(H)** (n = 7~9); **(I)** Mitochondria were isolated from mouse kidneys (n = 3); **(J)** Mitochondria Cdc42 expression was upregulated in CP-AKI mice (n = 3); **(K-M)** ZCL278 enhanced CP-induced increase in renal protein expression of mitophagy-related indicators Parkin and Pink1** (K)**, and counteracted CP-induced changes in mitochondrial fission-related indicator Drp1, mitochondrial fusion-related indicator Mfn2** (L)**, and mitochondrial biogenesis related indicators Pgc-1α and Nrf2 **(M)** (n = 6); **(N)** Representative TEM imaging revealed that ZCL278 alleviated mitochondrial ultrastructural damage in CP-induced AKI RTECs (red arrows: mitochondria, yellow arrows: brush border, scale bar = 1 μm, n  =  3); **(O)** Quantification of the mitochondrial aspect ratio; **(P)** ZCL278 restored renal ATP levels in CP-AKI mice (n = 7~9); **(Q-R)** ZCL278 inhibited the upregulation of Cdc42 and GTP-Cdc42 protein levels in I/R-induced AKI mice (n = 3 in control and sham groups, n=6 in I/R and ZCL+I/R+ZCL group); **(S-T)** ZCL278 attenuated I/R-induced elevation in BUN **(S)** and Cr levels **(T)** (n = 3 in control and sham groups, n=6 in I/R group, and n=10 in ZCL+I/R+ZCL group); **(U-W)** ZCL278 enhanced I/R-induced increase in renal protein expression of Parkin and Pink1 **(U)**, and reversed I/R-induced changes in Drp1 and Mfn2** (V)**, as well as Pgc-1α and Nrf2 **(W)** (n  =  3); Data were presented as means ± SD unless other noted. * 0.01 <* p* ≤ 0.05, ** 0.001< *p* ≤ 0.01, *** 0.0001 <*p* ≤ 0.001, **** p ≤ 0.0001.

**Figure 3:**
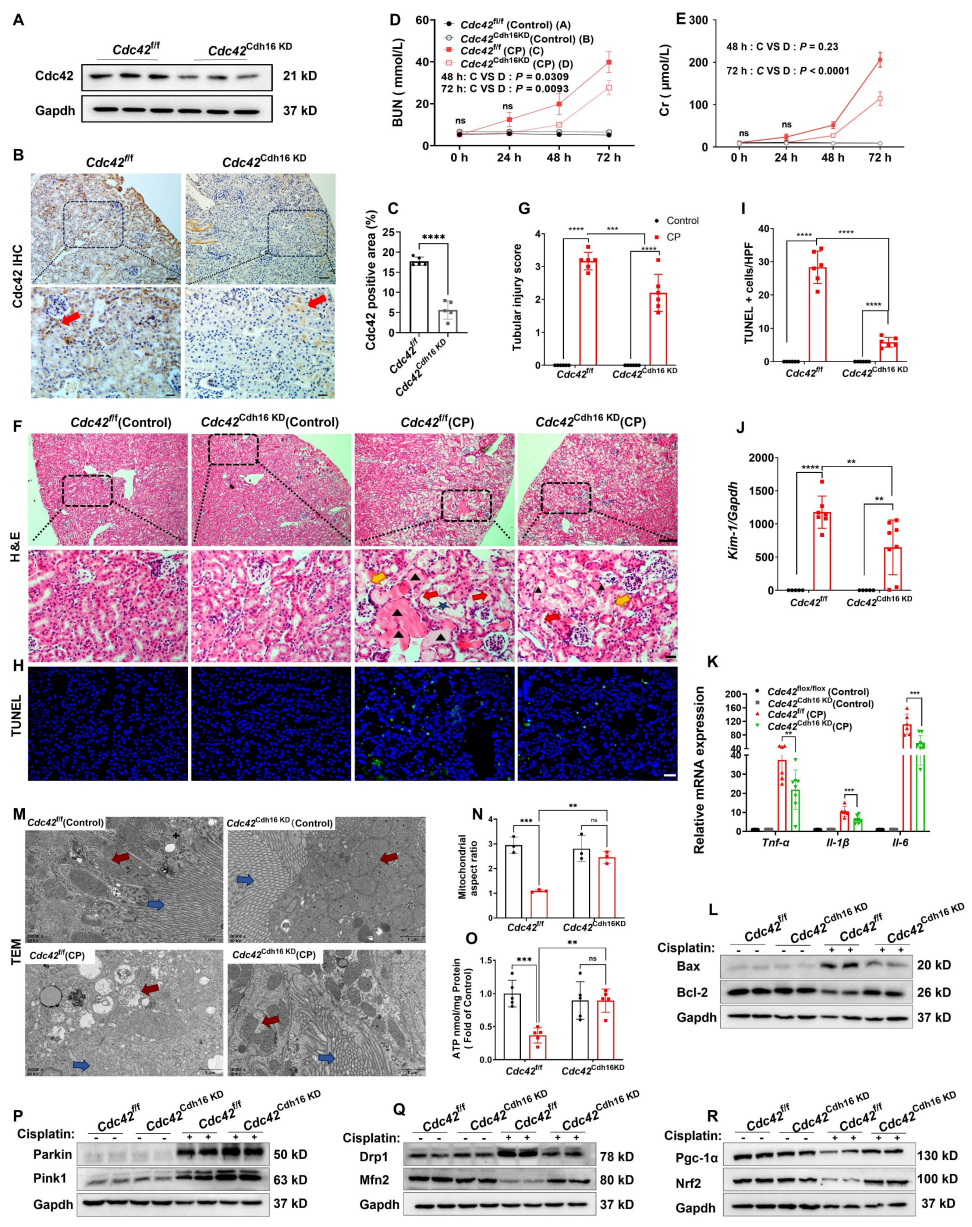
** Conditional knockdown of *Cdc42* in RTECs alleviated kidney injury and restored mitochondrial function in AKI mice. (A)** The knockdown effect of *Cdc42* was verified by WB;** (B-C)** IHC staining showed reduced Cdc42 expression in RTECs of *Cdc42*^Cdh16 KD^ mice; **(D-E)** BUN **(D)** and blood Cr levels **(E)** were lower in* Cdc42*^Cdh16 KD^ mice than those of *Cdc42*^f/f^ mice after CP administration (n = 8~10 in CP group, n = 5 in control group, means ± SEM); **(F)** H&E staining revealed milder renal damage in *Cdc42*^Cdh16 KD^ mice post-CP (scale bars: 100 μm, overview; scale bars: 20 μm, magnified; n = 5~6); **(G)** Analysis of renal tubular injury scores; **(H)**Representative images and quantification **(I)** of TUNEL staining in mouse kidney sections showed decreased apoptotic cells in *Cdc42* cKD mice after CP challenge (Green staining and blue staining indicated TUNEL-positive cells and nuclei, respectively; n = 5~6, scale bar = 20 μm); **(J-K)** mRNA expression of *Kim-1*** (J)** and pro-inflammatory factors **(K)** in *Cdc42*^Cdh16 KD^ mice was significantly lower than that in *Cdc42*^f/f^ mice when challenged with CP (n = 5~8); **(L)** Bax and Bcl-2 protein expression were reversed by *Cdc42*^Cdh16 KD^ upon CP exposure (n = 5~6); **(M)** Representative transmission electron micrographs of mitochondria from mice RTECs indicated improved mitochondrial ultrastructure in *Cdc42*^Cdh16 KD^ mice after CP exposure (red arrows: mitochondria, blue arrows: brush border; n = 3); **(N)** Mitochondrial aspect ratio quantification; **(O)** *Cdc42* cKD attenuated the decline in renal ATP levels of AKI mice (n = 5); **(P-R)** Protein expression of mitochondrial homeostasis related indicators showed *Cdc42* cKD improved mitochondrial homeostasis in AKI mice (n = 5~6). Data were presented as means ± SD unless other noted. * 0.01 <* p* ≤ 0.05, ** 0.001< *p* ≤ 0.01, *** 0.0001 <*p* ≤ 0.001, **** p ≤ 0.0001.

Mice were housed in a specific pathogen-free (SPF) facility under controlled temperature (22 ± 1 °C) and humidity (55 ± 10%), with a 12-hour light/dark cycle, and *ad libitum* access to autoclaved water and food. After a one-week acclimatization, mice were randomly assigned to experimental groups as detailed in Figures 1, 2, and 3. All experimental treatments were performed at consistent times of the day to minimize circadian influences on renal function.

### Measurement of blood levels of creatinine and urea

The blood levels of creatinine and urea in mice were measured using a high-performance liquid chromatography-tandem mass spectrometry (HPLC-MS/MS) as previously described 11.

### Plasmids, lentivirus short-hairpin RNA construction, and cell transfection

The stable knockout (KO) cell line was constructed using CRISPR-Cas9 technology. LentiCRISPRv2-sgRNA-*CDC42* (Sense 5'-CACCGACAGTCGGTACATATTCCGA-3' and anti-sense 5'-AAACTCGGAATATGTACCGACTGTC-3'), lentiCRISPRv2-sgRNA-*KLF2* (sense 5'-CACCGCGCGTCGTCGAAGAGACCGA-3' and anti-sense 5'-AAACTCGGTCTCTTCGACGACGCGC-3'), lentiCRISPRv2-sgRNA-*HIF-1α* (sense 5'-CACCGAGATGCGAACTCACATTATG-3' and anti-sense 5'-AAACCATAATGTGAGTTCGCATCTC-3'), lentiCRISPRv2-sgRNA-*PINK1* (sense 5'-CACCGCGTGGACCATCTGGTTCAAC-3' and anti-sense 5'-AAACGTTGAACCAGATGGTCCACGC-3'), and the lentiCRISPRv2-sgRNA-NC were purchased from Bio-rabbit (China, Shanghai). Lentivirus was packaged in HEK293T cells with Lipofectamine 3000 Transfection Reagent (Invitrogen, #L3000008) according to the manufacturer's instructions. After 48 h of infection, the supernatant of HEK293T cells was collected and used to infect HK-2 cells in the presence of polybrene (Beyotime; #C0351) to assist transfection. Transfected cells were then selected with puromycin (Beyotime; #ST551) to generate stable knockout cell lines. The knockout efficiency was evaluated by western blot (WB) analysis.

### RNA sequencing (RNA-seq)

RNA sequencing was performed by Sangon Biotech (China, Shanghai) following total RNA extraction from HK-2 cells using TRIzol reagent (Invitrogen, #15596018CN). For samples without biological replicates, read count data were normalized using the TMM method, followed by an analysis of variance analysis with DEGseq. For samples with biological replicates, differential expression analysis was conducted using DESeq. Significantly different genes were identified based on adjusted *p* value ≤ 0.05 and absolute log_2_ fold change (| log_2_FC |) ≥ 1.

### Statistical analysis

Data analysis was performed using SPSS 27.0 (SPSS Inc., Chicago, IL), and figures were generated with GraphPad Prism version 9 (La Jolla, CA). Normality was assessed using the Kolmogorov-Smirnov, Anderson-Darling, D'Agostino-Pearson omnibus, or Shapiro-Wilk test. Differences between two groups were analyzed using an independent-sample *t*-test, while One-way or two-way analysis of variance (ANOVA), followed by Tukey′s or Games-Howell post-hoc comparison, was used for multiple group comparisons. * *p* ≤ 0.05, ** 0.001 <* p* ≤ 0.01, ***0.0001 <* p* ≤ 0.001 and **** *p* ≤ 0.0001 were considered significant difference.

Additional experimental details are presented in the Supplementary Material.

## Results

### Human scRNA-seq data analysis identified *CDC42* as a pivotal gene in AKI pathogenesis

To explore the potential pathogenic mechanisms of AKI, we first downloaded raw data of human AKI from published scRNA-seq databases 24. After performing dimensionality reduction and clustering analysis (Figure 1A), and annotating cell types based on canonical markers (Figure 1B), we identified 12 major clusters from 96527 cells between control and AKI groups. Renal proximal tubule (PT) cells have become a major target for exploring AKI in recent years due to their abundance and heightened susceptibility/responsiveness to external insults 25,26. KEGG pathway enrichment analysis of PT cells from control and AKI groups revealed that differentially expressed genes (DEGs) were significantly enriched not only in infection-related pathways that associated with mortality etiology in AKI patients, but also significantly in actin cytoskeleton signaling pathways, which particularly captured our attention (Figure 1C).

The actin cytoskeleton, one of the most critical intracellular scaffold structures, maintains cell morphology, motility, and division through diverse actin isoforms and regulatory proteins governed by intricate signaling networks 27,28. Researches have demonstrated its involvement in mitochondrial homeostasis regulation 29. Its assembly/disassembly is primarily regulated by the Rho GTPases family, consisting of RHOA, RAC1, and CDC42, which coordinates different cellular processes through downstream effectors 30. Further volcano analysis in PT cells of control and AKI groups revealed that *CDC42* is the most significantly altered gene in Rho GTPases family (Figure 1D). Violin plot also showed that* CDC42* expression was significantly upregulated in PT cells of the AKI group (Figure 1E). Taken together, the scRNA-seq data analysis of human AKI suggested *CDC42* may be a pivotal gene involved in the pathogenesis of AKI.

### CDC42 was upregulated in cisplatin (CP)- and ischemia-reperfusion (I/R)-induced AKI mouse models

To further define the role of CDC42 in AKI, we adopted two most recognized and widely used murine models of CP- and I/R-induced AKI 31. In CP-induced AKI mice, blood urea nitrogen (BUN, Figure 1F) and creatinine (Cr, Figure 1G) were progressively increased and climaxed at 72 h post-exposure. Histologic analysis of kidney sections from the AKI mice (72 h post CP exposure) revealed severe tubular injury, as evidenced by hematoxylin-eosin (H&E) staining (Figure 1H and 1I). Immunohistochemistry (IHC) analysis showed that Cdc42 was abundantly expressed in mice kidney tissues, particularly in the renal tubular epithelial cells (RTECs) (including both PT cells and distal convoluted tubule (DCT) cells) (Figure 1J), which was consistent with the human AKI scRNA-seq results (Figure 1E and Supplementary Figure S1). Moreover, both IHC (Figure 1K) and q-PCR (Figure 1L) analyses indicated a significant upregulation of Cdc42 in the kidneys of CP-AKI mice. In I/R-induced AKI mice, renal injury was evidenced by significantly elevated BUN and blood Cr levels, and severe tubular damage (Figure 1M-1P). In addition, no significant difference in BUN, Cr, or tubular injury scores were observed between the control and the sham groups, suggested that surgical stress did not affect renal function. Similarly, IHC staining and q-PCR analyses revealed a significantly increased Cdc42 expression in the RTECs of I/R-AKI mice when compared with the sham controls (Figure 1Q-1S).

### Cdc42 inhibition protected against kidney injury in CP-induced AKI mice

To investigate the therapeutic potential of Cdc42 inhibition in AKI, we administered ZCL278, a Cdc42-selective inhibitor, to AKI mice 32. In CP-induced AKI mouse model, two treatment regimens were designed: 1) post-treatment (CP+ZCL) to evaluate therapeutic efficacy, and 2) combined pre- and post-treatment (ZCL+CP+ZCL) to assess preventive and therapeutic efficacy. Notably, ZCL278 treatment effectively reduced renal Cdc42 protein expression (Figure 2A) and active GTP-Cdc42 levels (Figure 2B), significantly lowering elevated BUN and blood Cr concentrations in CP-AKI mice (Figure 2C and 2D). Importantly, ZCL278 showed no nephrotoxicity in healthy mice, as evidenced by stable BUN and Cr levels (Figure 2C and 2D). Furthermore, ZCL278 treatment prolonged the survival time of CP-induced AKI mice, with a median survival time of 108 hours in the CP group and 132 hours in the CP+ZCL group, indicating that ZCL278 has a sustained protective effect (Figure 2E). Meanwhile, H&E and TUNEL staining analyses (Supplementary Figure S2A-S2D), protein levels of Bax (a pro-apoptotic regulator) and Bcl-2 (an anti-apoptotic regulator) (Figure 2F), mRNA expression of kidney-injury-molecule-1 (*Kim-1*) and inflammatory cytokines (*Il-1β*, *Il-6*, and *Tnf-α*) (Figure 2G-2H) indicated that ZCL278 greatly attenuated kidney injury in CP-induced AKI mice. Given the concordance between mitochondrial (Supplementary Figure S3) and whole-cell (Figure 2F) Bax expression under CP challenge with or without ZCL278 treatment, subsequent studies used whole-cell Bax expression as an apoptotic marker.

### Cdc42 inhibition alleviated mitochondrial dysfunction in CP-AKI mice

Cellular oxidative stress, induced by ROS released from mitochondrial dysfunction, is the central pathological mechanism in AKI 6,7,10. Given the crosstalk between actin cytoskeleton dynamics and mitochondrial function 29, we hypothesize that Cdc42 inhibition may protect against AKI by modulating mitochondrial function. Isolation of mitochondria from mouse kidneys (Figure 2I-2J) revealed that Cdc42 was increased in CP-AKI mouse kidney mitochondria, which indicating its involvement in AKI mitochondrial regulation. Mitochondria homeostasis relies on a dynamic balance among PGC-1α/NFR2-mediated mitochondrial biogenesis, MFN2/DRP1-mediated mitochondrial fusion and fission, and PINK1/PARKIN-mediated mitophagy 9, with impairment of any of these processes causing mitochondrial dysfunction. Notably, ZCL278 treatment effectively maintained mitochondrial homeostasis in CP-induced AKI mice (Figure 2K-2M). To further assess mitochondrial function, we characterized mitochondrial ultrastructure in CP-AKI mice with or without ZCL278 treatment. As illustrated in Figure 2N and 2O, Cdc42 inhibition with ZCL278 treatment significantly improved CP exposure-caused mitochondrial ultrastructural abnormalities. In addition, ZCL278-mediated Cdc42 inhibition rescued the compromised ATP generation (Figure 2P) in CP-challenged mice.

Taken together, pharmacological inhibition of Cdc42 via ZCL278 treatment restored mitochondrial function in CP-induced kidney injury. Both administration routes of ZCL278 provided protective effects, with combined pre- and post-treatment showing superior efficacy.

### Cdc42 inhibition protected against kidney injury and mitochondrial dysfunction in I/R-induced AKI

To comprehensively evaluate the reno-protective effects of Cdc42 inhibition across etiologically distinct AKI models, we assessed renal injury and mitochondrial function in I/R-AKI mice following ZCL278 treatment. Consistent with results in CP-AKI mice, ZCL278 reversed I/R-induced increase in Cdc42 protein expression and its activation levels (Figure 2Q-2R), reduced elevated BUN and blood Cr concentrations (Figure 2S and 2T), and alleviated renal tubular injury (Supplementary Figure S2E and S2F). Additionally, ZCL278 treatment effectively preserved mitochondrial function (Figure 2U-2W).

Given the comparable efficacy in both CP- and I/R-AKI mouse models, combined with the higher clinical relevance of nephrotoxic AKI and the mouse mortality associated with I/R surgery, we primarily conducted subsequent animal experiments in CP-induced AKI model.

### Conditional knockdown of *Cdc42* in RTECs alleviated kidney injury and mitochondrial dysfunction in CP-induced AKI mice

To establish an appropriate renal conditional *Cdc42* knockdown (cKD) mouse model to mimic CP-induced kidney injury, we first analyzed publicly available scRNA-seq data from control and CP-AKI mice 33 (Supplementary Figure S4A-S4B). The results revealed that the *Nagl* (a marker of AKI) 34,35 (Supplementary Figure S4C-S4E) and *Cdc42* (Supplementary Figure S4F-S4H) expression in both PT and DCT cells were significantly increased in CP-AKI mice, with DCT cells showing injury severity similar to or greater than that of PT cells. CP exposure is known to impair glomerular filtration rate (GFR) primarily by damaging the distal nephron including DCT, connecting tubule and collecting ducts, and the S3 segment of the PT 36,37. This understanding is supported by previous studies 38,39 and our H&E staining (Figure 1H), which demonstrate that CP causes damage to both PT and DCT cells in AKI mice. Based on these findings, we selected *Cdh16*-Cre mice to generate a RTEC-specific *Cdc42* conditional knockdown mouse model using the Cre-LoxP system, enabling targeted investigation of Cdc42 function in CP-AKI. The knockdown efficiency was verified by identification of mice genotyping (Supplementary Figure S5), WB assay (Figure 3A) and IHC staining (Figure 3B-3C).

Consistent with the nephro-protective profile of ZCL278 treatments, *Cdc42* cKD in mice RTECs attenuated CP-induced increases in BUN and Cr levels (Figure 3D-3E), ameliorated histopathological damage (Figure 3F-3G), and decreased renal apoptotic cells in CP-AKI mice (Figure 3H-3I). Furthermore, inflammatory and apoptotic markers further supported these findings, as *Cdc42* cKD mice exhibited lower Bax, *Kim-1*, *Il-6*, *Tnf-α*, and *Il-1β*, alongside higher Blc-2 expression (Figure 3J-3L). Further analyses revealed that *Cdc42* cKD AKI mice exhibited improved mitochondrial morphology (Figure 3M-3N), significantly higher ATP levels (Figure 3O) and restored mitochondrial homeostasis (Figure 3P-3R).

Taken together, these results revealed that pharmacological and genetic blockade of Cdc42 improved mitochondrial dysfunction, contributing to its protection against AKI.

### Inhibition of CDC42 suppressed CP-induced cell injury and improved mitochondrial dysfunction* in vitro*

**Figure 4:**
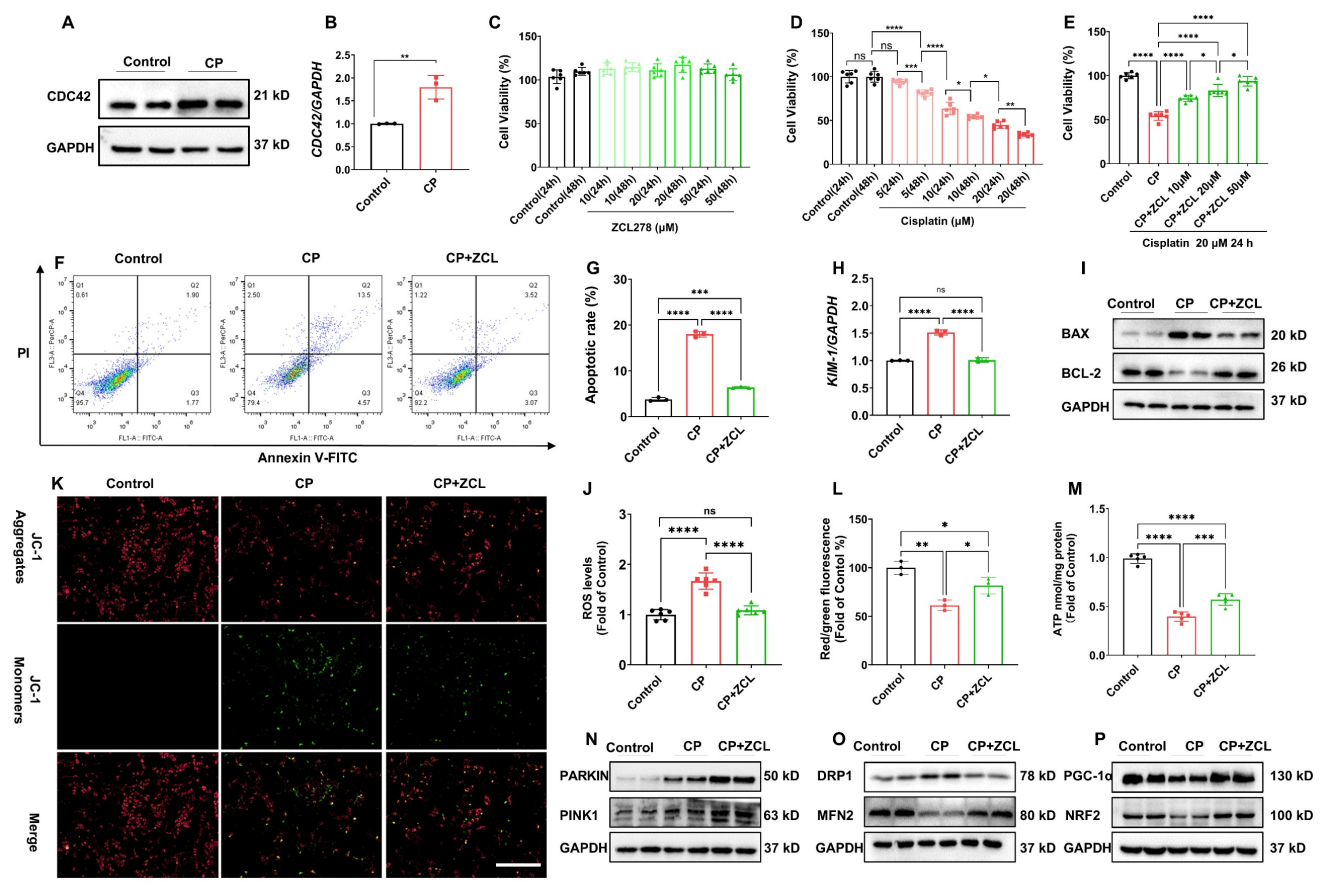
** ZCL278 treatment attenuated CP-induced cell injury and mitochondrial dysfunction *in vitro*. (A-B)** The protein **(A)** and mRNA **(B)** levels of CDC42 were significantly increased in HK-2 cells upon CP exposure (n = 3); **(C)** ZCL278 at tested conditions had a negligible effect on cellular activity (n = 6); **(D)** CP exposure reduced HK-2 cells cell viability in a concentration- and dose-dependent manner (n = 6);**(E)** ZCL278 concentration-dependently ameliorated the CP-induced decline in cell viability (n = 6); **(F)** Representative images of apoptosis by flow cytometry showed ZCL278 reduced CP-induced cell apoptosis (n = 3); **(G)** Quantitative results of** (F)**;** (H)** ZCL278 alleviated CP-induced upregulation of *KIM-1*mRNA (n = 3); **(I)** ZCL278 reversed CP-induced changes in protein expression of BAX and BCL-2 (n = 3); **(J)** ZCL278 reduced CP exposure-induced increase in cellular ROS accumulation (n = 3); **(K-L)** ZCL278 alleviated CP-induced decline in cellular MMP (n = 3); **(M)** ZCL278 mitigated the decline in ATP levels in CP-treated cells (n = 3); **(N-P)** ZCL278 reversed CP exposure-caused changes in protein expression of DRP1 and MFN2** (O)**, PGC-1α and NRF2 **(P)**, and enhanced CP-caused increase in protein expression of PARKIN and PINK1 in HK-2 cells **(N)** (n = 3). Data were presented as means ± SD. * 0.01 <* p* ≤ 0.05, ** 0.001< *p* ≤ 0.01, *** 0.0001 <*p* ≤ 0.001, **** p ≤ 0.0001.

We employed HK-2 cells, an immortalized RTEC as an *in vitro* model to investigate the direct role of CDC42 in AKI. In accordance with our *in vivo* findings, CDC42 mRNA and protein expression was significantly upregulated in CP-treated HK-2 cells compared with the controls (Figure 4A-4B). CCK-8 assays showed that ZCL278 treatment (up to 50 μM for 48 h) did not affect cell viability (Figure 4C), indicating its safety profile. As anticipated, CP exposure decreased cell viability concentration- and time-dependently (Figure 4D), with 20 μM CP for 24 h reducing viability to ~50% of control levels. These conditions were set for subsequent experiments to mimic AKI *in vitro*. Notably, ZCL278 restored CP-induced viability loss in a concentration-dependent manner (Figure 4E), accompanied by reduced apoptosis (Figure 4F-4G) and* KIM-1* expression (Figure 4H). Additionally, ZCL278 treatment reversed CP-induced changes in BAX and BCL-2 protein levels (Figure 4I).

Given that mitochondrial dysfunction is a primary source of ROS in AKI 6,7,10, we assessed ROS levels and found that ZCL278 treatment significantly reduced CP-induced ROS accumulation (Figure 4J). Furthermore, ZCL278 treatment restored ATP synthesis and mitochondrial membrane potential (MMP) (Figure 4K-4M), while WB analyses of mitochondrial homeostasis markers further confirmed that CDC42 inhibition mitigated CP-induced mitochondrial dysfunction (Figure 4N-4P).

### Genetical knockout of *CDC42* protected HK-2 cells against CP-caused cell injury and mitochondrial dysfunction

**Figure 5:**
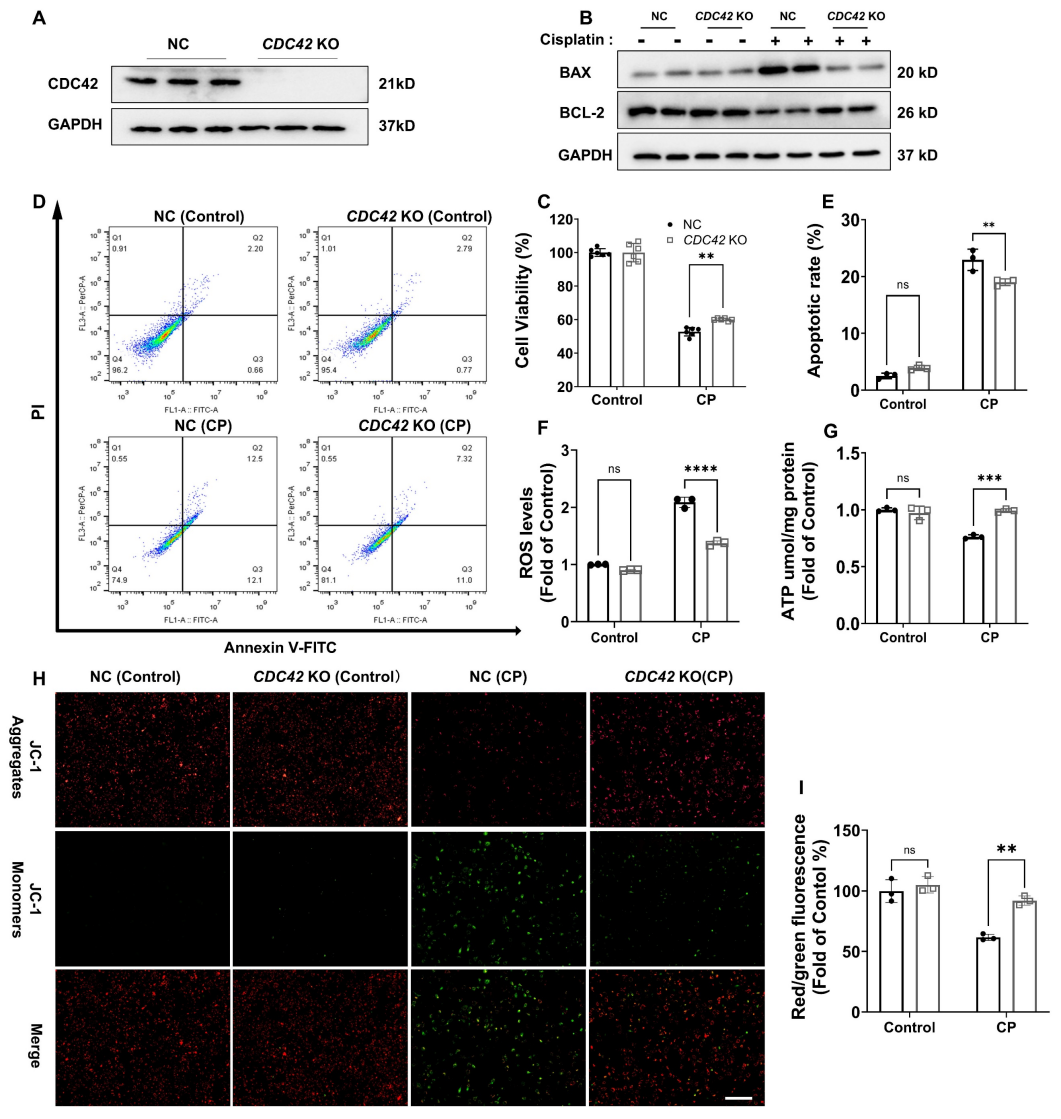
** Knockout of *CDC42* in HK-2 cells protected against CP-induced cell injury and mitochondrial dysfunction *in vitro*. (A)** *CDC42* knockout effect in HK-2 cells was verified by WB;** (B)** *CDC42* KO reversed CP-induced elevation of BAX and reduction of BCL-2 protein expression (n = 3); **(C)** *CDC42* KO alleviated CP-induced decrease in cell viability (n = 6); **(D-E)** Flow cytometry analysis indicated that *CDC42* KO reduced CP-induced cell apoptosis (n = 3); **(F)** *CDC42* KO reduced cellular ROS accumulation upon CP exposure (n = 3); **(G)** *CDC42* KO alleviated CP-induced decrease in cellular ATP levels (n = 3); **(H-I)** Representative images of the mitochondrial membrane potential **(H)** and the related quantitative analyses of JC-1 fluorescence intensity** (I)** demonstrated that *CDC42* KO reversed the decrease in MMP after CP challenge (n = 3, scale bar = 200 μm). Data were presented as means ± SD. * 0.01 <* p* ≤ 0.05, ** 0.001< *p* ≤ 0.01, *** 0.0001 <*p* ≤ 0.001, **** p ≤ 0.0001.

To further validate the protective role of CDC42 inhibition in AKI, we constructed a stable *CDC42* knockout (KO) HK-2 cell line (Figure 5A). WB analysis revealed that *CDC42* KO reversed CP-induced changes in BAX and BCL-2 protein expression in the negative control (NC) cells (Figure 5B). Also, *CDC42* KO alleviated the CP-induced decline in cell viability (Figure 5C) and CP-induced increase in cell apoptosis (Figure 5D-5E). Additionally, *CDC42* KO markedly mitigated mitochondrial dysfunction and alleviated cellular oxidative stress, as evidenced by a marked reduction in CP-induced ROS accumulation (Figure 5F) and restoration of MMP and ATP levels (Figure 5G-5I).

Taken together, these above *in vitro* cellular studies showed that inhibition of CDC42 protected HK-2 cells from ROS-induced cellular oxidative stress injury by ameliorating mitochondrial dysfunction, which further supported CDC42 as a potential therapeutic target for AKI.

### *KLF2* was a downstream target gene of CDC42 in regulating AKI

**Figure 6:**
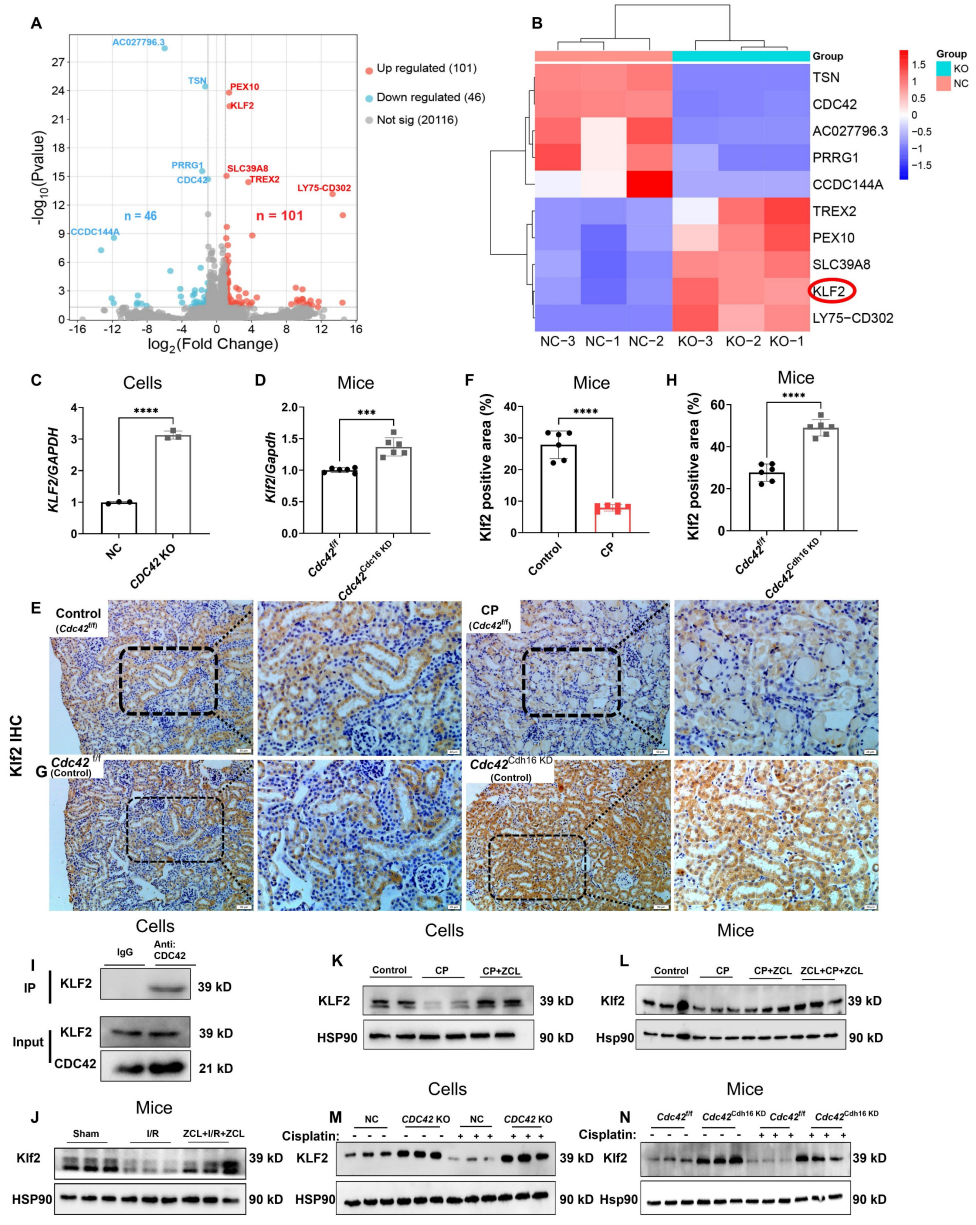
***KLF2* was the downstream target of CDC42 in AKI. (A)** Volcano plots of the detected genes between NC cells and *CDC42* KO cells (n = 3); **(B)** Heatmap showed 10 DEGs with the most significant differences; **(C)** *CDC42* KO increased the mRNA expression of *KLF2* in HK-2 cells (n = 3);** (D)** Renal *Klf2* mRNA expression was increased in *Cdc42*^Cdh16 KD^ mice compared to *Cdc42*^f/f^ mice (n = 5); **(E-F)** Immunohistochemistry showed reduced expression of Klf2 in AKI mice (scale bars: 50 μm, overview; scale bars:20 μm, magnified; n = 6); **(G-H)** Immunohistochemistry revealed increased Klf2 expression in *Cdc42*^Cdh16 KD^ mice compared to *Cdc42*^f/f^ mice (scale bars: 50 μm, overview; scale bars:20 μm, magnified; n = 5); **(I)** Co-IP assays confirmed the interaction between CDC42 and KLF2 in HK-2 cells (n = 3); **(J)** Decreased Klf2 protein expression in I/R-induced AKI mice was reversed by ZCL278 (n = 3 in sham group, n = 6 in other groups); **(K)** ZCL278 reversed CP-induced reduction of KLF2 protein expression in HK-2 cells (n = 3); **(L)** ZCL278 ameliorated CP-induced reduction in renal Klf2 protein in mice (n = 6); **(M)** *CDC42* KO increased the protein expression of KLF2 in HK-2 cells (n = 3); **(N)** Renal Klf2 protein expression was upregulated in *Cdc42*^Cdh16 KD^ mice when compared to *Cdc42*^f/f^ mice (n = 3). Data were presented as means ± SD. * 0.01 <* p* ≤ 0.05, ** 0.001< *p* ≤ 0.01, *** 0.0001 <*p* ≤ 0.001, **** p ≤ 0.0001.

To elucidate the molecular mechanisms underlying CDC42-mediated renal protection in AKI, we performed RNA sequencing (RNA-seq) on *CDC42* KO cells and NC cells. A total of 147 DEGs were identified (Figure 6A), while heatmap analysis (Figure 6B) highlighted the top 10 most significantly altered genes, among which *KLF2* emerging as a key candidate due to its well-established roles in mitochondrial function 40,41, oxidative stress-mediated cell damage and apoptosis 42. This hypothesis was then supported by q-PCR, showing that both* CDC42* KO (*in vitro*) and cKD (*in vivo*) significantly increased *KLF2* mRNA levels (Figure 6C-6D). Furthermore, IHC staining revealed abundant Klf2 expression in RTECs, which was decreased following CP exposure (Figure 6E-6F), but was restored in *Cdc42*^Cdh16 KD^ mice (Figure 6G-6H).

**Figure 7:**
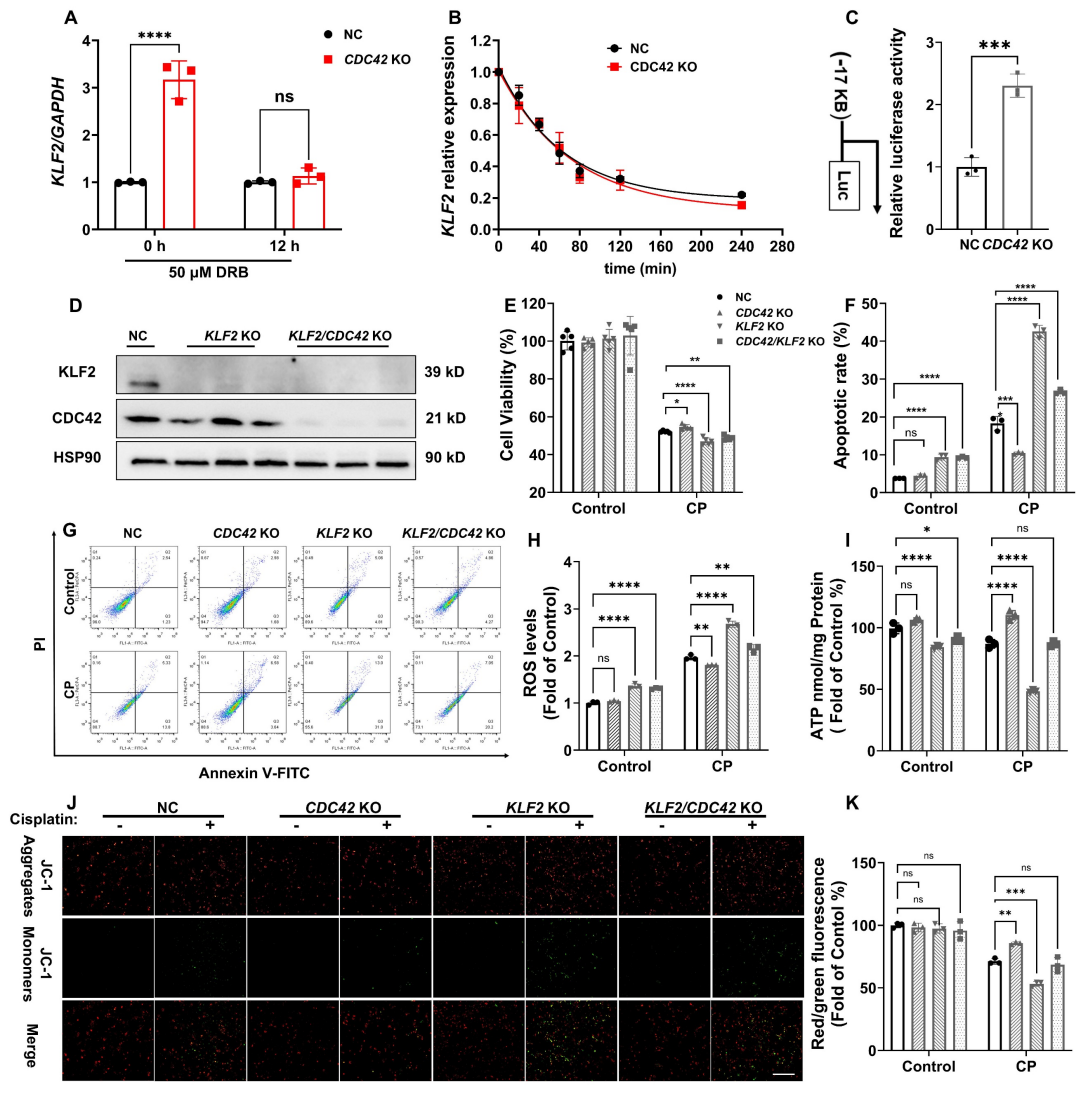
***CDC42* KO protected CP-induced cell injury by enhancing *KLF2* promoter activity. (A)** mRNA induction of *KLF2* by *CDC42* KO was abrogated after 50 μM DRB treatment (n = 3);** (B)** mRNA stability showed that the mRNA half-life of *KLF2* was similar in NC cells and *CDC42* KO cells (n = 3);** (C)** The dual-luciferase assay revealed a significant increase in *KLF2* promoter activity in *CDC42* KO cells (n = 3);** (D)** The knockout effect of *CDC42* and *KLF2* in HK-2 cells was verified by WB; **(E)** The protection of *CDC42* KO in cell viability was counteracted by *KLF2* KO upon CP exposure (n = 5); **(F-G)** Representative images of apoptosis assay by flow cytometry** (G)** and the statistical analysis **(F)** indicated that *KLF2* KO aggravated CP-induced cell apoptosis (n =3);** (H)** *KLF2* KO in HK-2 cells significantly increased cellular ROS concentrations (n = 3); **(I)** *KLF2* KO worsened the CP-induced decline in cellular ATP levels (n = 3); **(J-K)** Representative images of the mitochondrial membrane potential **(J)** and the quantitative analyses of JC-1 fluorescence intensity (red/green) **(K)** revealed that *KLF2* KO further contributed to the decrease in MMP after CP exposure (n = 3, scale bar: 200 μm). Data were presented as means ± SD. * 0.01 <* p* ≤ 0.05, ** 0.001< *p* ≤ 0.01, *** 0.0001 <*p* ≤ 0.001, **** p ≤ 0.0001.

Critically, co-immunoprecipitation (Co-IP) assays indicated the protein interaction between CDC42 and KLF2 (Figure 6I). Importantly, CDC42 inhibition reversed the CP- and I/R-induced reduction in KLF2 expression in both *in vivo* (Figure 6J, 6L, 6N) and *in vitro* (Figure 6K, 6M) models, consistent with IHC results. This regulation axis was also supported by the fact *KLF2* KO failed to affect the CDC42 expression (Figure 7D).

These findings identify KLF2 as a critical downstream target of CDC42 inhibition, providing novel insights into its protective role against AKI in mitochondria and oxidative stress.

### *CDC42* KO increased *KLF2* promoter activity and exerted its protective effect against kidney injury by mediating KLF2

To explore the mechanism underlying the regulation of *KLF2* by CDC42, we examined *KLF2* expression in the presence and absence of DRB (a potent RNA polymerase inhibitor). Notably, the increased *KLF2* mRNA level caused by *CDC42* KO was completely abolished by DRB treatment (50 μM) (Figure 7A), indicating that CDC42-mediated KLF2 regulation occurs at the transcriptional level. Considering the mature mRNA abundance is influenced by both transcription rate and degradation 43, we first assessed the half-life of *KLF2* mRNA in HK-2 cells. We observed that *CDC42* KO caused a slight increase in *KLF2* mRNA stability (NC: *t_1/2_* = 43.6 ± 7.8 min vs CDC42 KO: *t_1/2_* = 50.2 ± 11.1 min, *p* = 0.38) (Figure 7B), demonstrating that CDC42-mediated *KLF2* primarily through transcriptional activation rather than mRNA stability. Then, dual-luciferase assay revealed that *CDC42* KO significantly increased the *KLF2* promoter activity (1.7-kB* KLF2*- Luc) by 2.5-fold, indicating that *CDC42* KO promotes *KLF2* transcription to increase its mRNA expression (Figure 7C).

To validate that CDC42 inhibition attenuates AKI via KLF2, we established *KLF2* KO and *KLF2/CDC42* double KO (dKO) cells (Figure 7D). Interestingly, *KLF2* KO alone significantly aggravated the CP-induced decrease in cell viability and increase in cell apoptosis. Unexpectedly, the protective effects of *CDC42* KO on CP-induced cell injury were virtually counteracted in *CDC42/KLF2* dKO cells (Figure 7E-7G). Consistently, *KLF2* KO exacerbated CP-induced mitochondrial dysfunction, as evidenced by increased cellular ROS concentrations, and decreased ATP and MMP levels. Strikingly, the ability of *CDC42* KO to restore mitochondrial function was nearly abolished in *CDC42/KLF2* dKO cells (Figure 7H-7K).

Collectively, these results show CDC42 exerts its protective effects against AKI by transcriptionally upregulating *KLF2*.

### KLF2 regulated AKI mechanism through transcriptional regulation of *HIF-1α*

**Figure 8:**
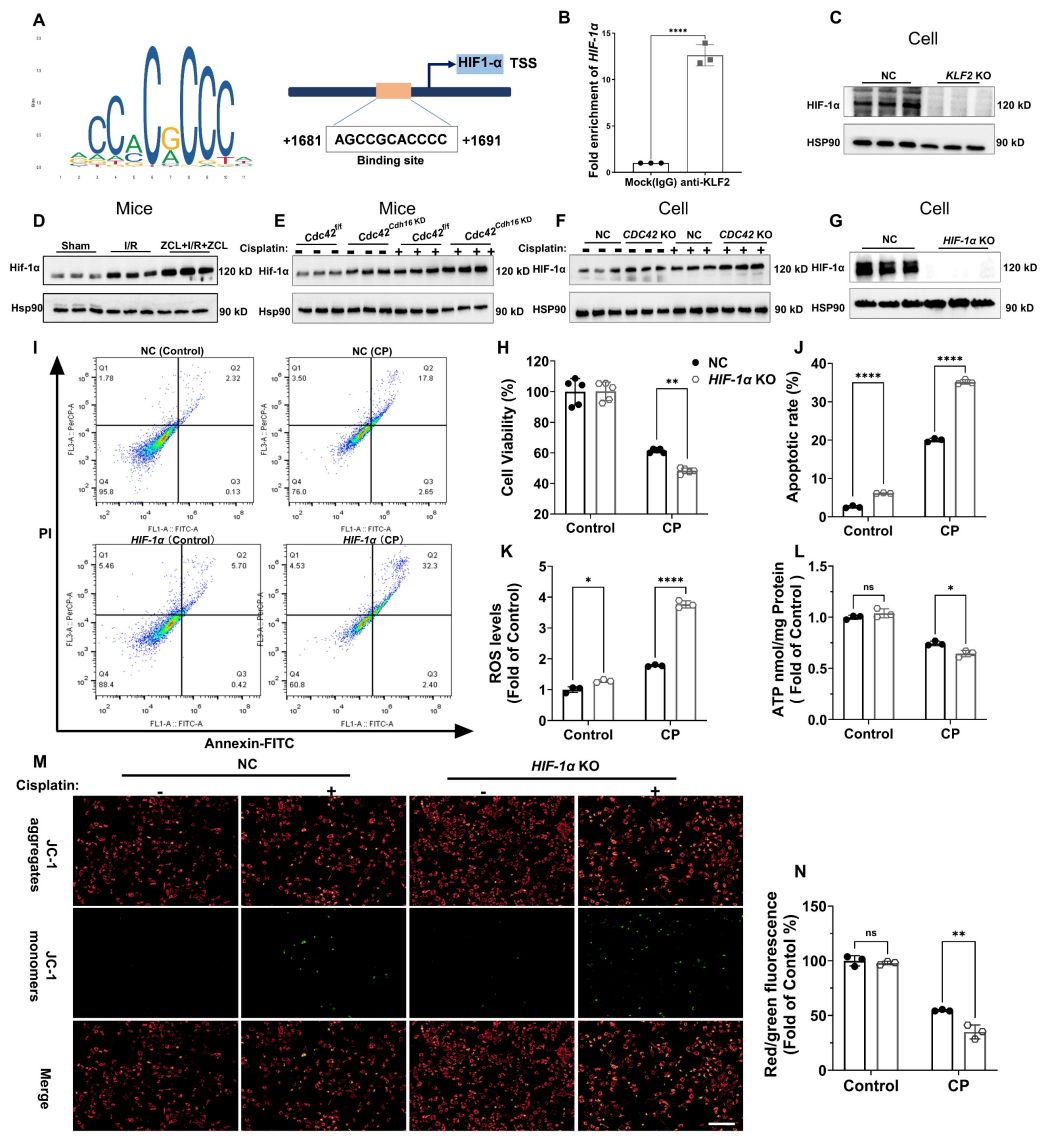
***HIF-1α* was the downstream target of KLF2 to regulate CP-induced cell damage and mitochondrial dysfunction *in vitro*. (A)** Binding sites of KLF2 in the* HIF-1 α* promoter region were predicted using the JASPAR database; **(B)** ChIP experiments validated the transcriptional regulation of* HIF-1a* by KLF2 (n = 3); **(C)** *KLF2* KO reduced HIF-1α protein expression in HK-2 cells (n = 3);** (D)** Renal Hif-1α protein expression was increased in I/R induced AKI mice and further increased after ZCL278 treatment (n = 3); **(E)** Renal Hif-1α protein expression was increased in *Cdc42*^Cdh16 KD^ mice compared with *Cdc42*^f/f^ mice (n = 3); **(F)** *CDC42* KO increased HIF-1α protein expression in HK-2 cells (n = 3); **(G)** The knockout efficiency of* HIF-1α* in HK-2 cells was verified by WB (n = 3); **(H)** *HIF-1α* KO increased CP-induced decrease in cell viability compared to NC cells (n = 5); **(I-J)** Representative images of flow cytometry** (I)** and the related statistical analysis **(J)** showed that *HIF-1α* KO increased CP-induced cell apoptosis (n = 3);** (K)** *HIF-1α* KO increased the ROS accumulation in HK-2 cells (n = 3); **(L)** *HIF-1α* KO aggravated CP-induced reduction in cellular ATP levels cells (n = 3);** (M-N)** Representative images of the mitochondrial membrane potential** (M)** and the quantitative analyses of JC-1 fluorescence intensity** (N)** demonstrated that *HIF-1α* KO exacerbated the CP-induced decrease in MMP (n = 3, scale bar = 200 μm). Data were presented as means ± SD. * 0.01 <* p* ≤ 0.05, ** 0.001<* p* ≤ 0.01, *** 0.0001 <*p* ≤ 0.001, **** *p* ≤ 0.0001.

To elucidate the mechanistic link between KLF2 and AKI, we performed a transcription factor database search using JASPAR, TRRUST, and KnockTF, identifying hypoxia-inducible factor 1α (HIF-1α) as a key downstream target. *HIF-1α* encodes the alpha subunit of transcription factor hypoxia-inducible factor-1 (HIF-1), a key regulator of cellular and systemic homeostatic responses to hypoxia 44. JASPAR database revealed conserved KLF2-binding motifs in the *HIF-1α* promoter (Figure 8A), which was experimentally validated by chromatin immunoprecipitation (ChIP) showing significant *HIF-1α* promoter enrichment in anti-KLF2 group (Figure 8B). Functionally, *KLF2* KO reduced HIF-1α protein expression (Figure 8C), whereas CDC42 inhibition via ZCL278 or genetic approaches (KO/cKD) upregulated HIF-1α in both CP- and I/R-AKI models (Figure 8D-8F).

To define HIF-1α' role in AKI, we generated *HIF-1α* KO cells and their corresponding rescue model (*HIF-1α* KO + *HIF-1α* cells) (Figure 8G and Supplementary Figure S6). As expected, *HIF-1α* KO worsened CP-induced cellular damage (Figure 8H-8J) and mitochondrial dysfunction (ROS↑, ATP↓, MMP↓; Figure 8K-8N), whereas the aforementioned damage was reversed in* HIF-1α* KO + *HIF-1α* cells (Supplementary Figure S6). These data collectively show that CDC42 inhibition ameliorates AKI through KLF2-dependent transcriptional activation of *HIF-1α.*

### HIF-1α mediated mitochondrial function via transcriptional regulation of *PINK1* in CP-induced AKI

Our study revealed that *HIF-1α* KO disrupts mitochondrial function, consistent with previous findings 45,46. To elucidate the molecular mechanism underlying HIF-1α's role in mitochondrial homeostasis, we investigated whether mitochondrial biogenesis, fusion/fission, or mitophagy is the primary regulatory process by which CDC42 inhibition against AKI.

**Figure 9:**
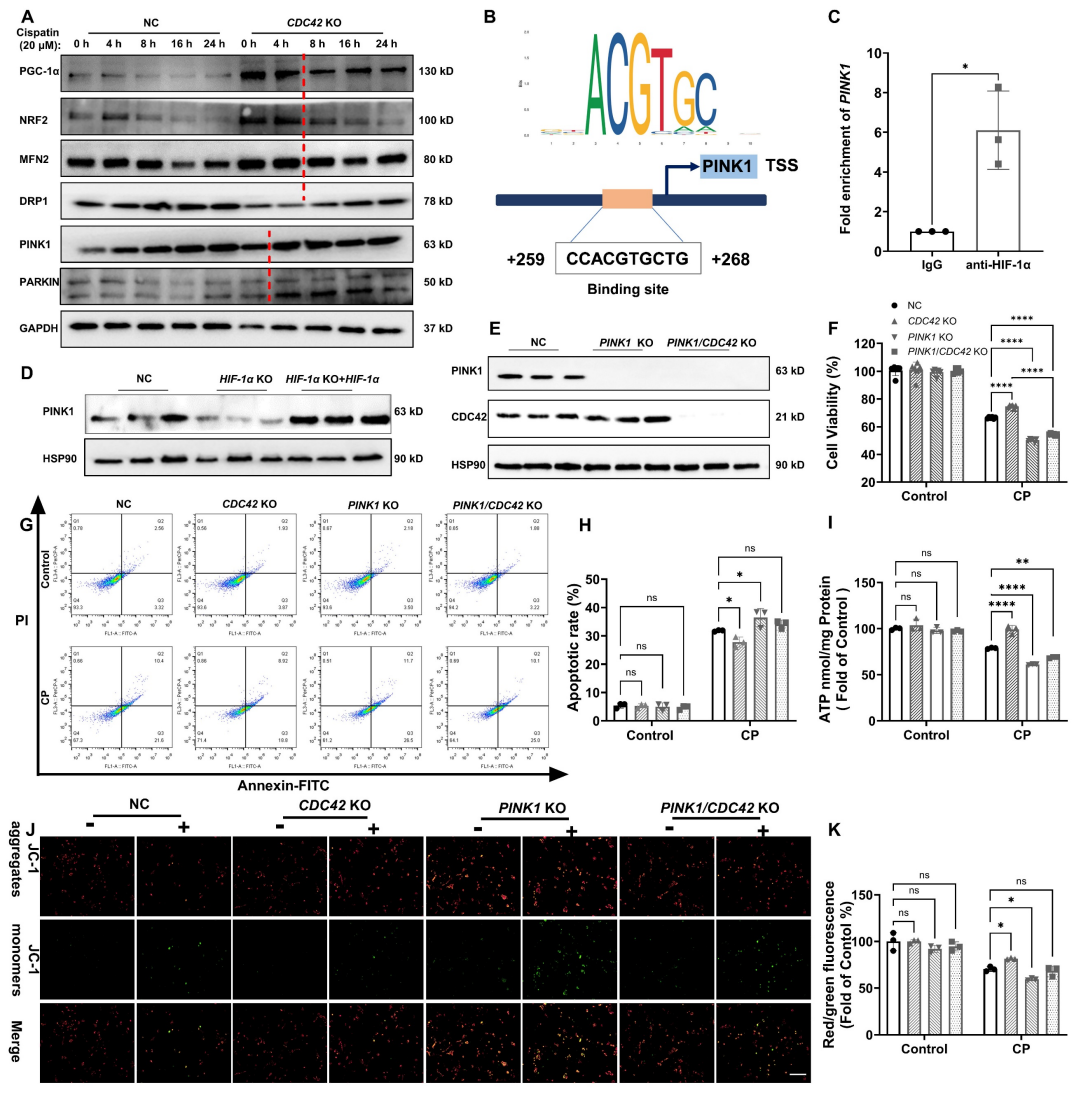
** CDC42 inhibition improved mitochondrial function to protect against AKI by targeting PINK1. (A)** A time course of mitochondrial homeostasis related proteins in NC and *CDC42* KO cells showed that mitophagy (PINK1, PARKIN) might be the leading process among CP-induced mitochondrial dysfunction (n = 3); **(B)** Binding sites of HIF-1α in the *PINK1* promoter region were predicted using the JASPAR database;** (C)** ChIP assays showed that HIF-1α transcriptionally regulated *PINK1* (n = 3); **(D)** Re-expression of *HIF-1α* in *HIF-1α* KO cells restored the decline in PINK1 protein levels (n = 3);** (E)** *PINK1* KO efficiency was verified in *PINK1* KO cells and *PINK1/CDC42* dKO cells (n = 3);** (F-H)** Protective effects of *CDC42* KO in cell viability **(F)** (n = 6) and cell apoptosis **(G-H)** were counteracted by *PINK1* KO (n = 3); Protective effects of *CDC42* KO in CP-induced mitochondrial dysfunction were counteracted by* PINK1* KO, as evident by mitochondrial ATP levels** (I)** and MMP levels (**J-K**, scale bar: 200 μm) (n = 3). Data were presented as means ± SD. * 0.01 <* p* ≤ 0.05, ** 0.001<* p* ≤ 0.01, *** 0.0001 <*p* ≤ 0.001, **** *p* ≤ 0.0001.

Time-course analysis of mitochondrial-related proteins in *CDC42* KO and NC cells exposed to CP revealed that mitophagy-related proteins (PINK1, PARKIN) rapidly increased at 4 h, while mitochondrial biogenesis (PGC-1α, NRF2) and fusion/fission (MFN2, DRP1) proteins showed significant changes at 8 h (Figure 9A). This suggests that mitophagy is the initial step in mitochondrial regulation upon CDC42 inhibition. Using the JASPER database, we identified* PINK1* as the most likely downstream gene of HIF-1α in mitochondrial-related genes (Supplementary Figure S7), validated by ChIP assay showing HIF-1α binding to the *PINK1* promoter (Figure 9B-9C) and WB results indicating PINK1 expression is positively regulated by HIF-1α (Figure 9D). Collectively, these results support the conclusion that mitophagy is the key initial process in CDC42/KLF2/HIF-1α-mediated mitochondrial regulation.

PINK1 is a crucial sensor of mitochondrial damage, initiating mitophagy to protect cells from external stimulus-induced mitochondrial dysfunction 47. To confirm that CDC42 inhibition provided the protective effect against AKI through regulating KLF2/HIF-1α/PINK1 axis, we examined the impact of CP exposure in *PINK1* KO cells and* PINK1/CDC42* dKO cells (Figure 9E). As expected, *PINK1* KO further exacerbated CP-induced cell injury and mitochondrial dysfunction, and more importantly, *PINK1* KO inhibited the protective effect of *CDC42* KO against CP-AKI *in vitro* (Figure 9F-9K). These findings suggest that PINK1 plays a crucial role in CDC42-mediated mitochondrial protection, supporting the hypothesis that CDC42 inhibition mitigates AKI by promoting mitophagy through KLF2/HIF-1α/PINK1 regulatory axis.

## Discussion

**Figure 10:**
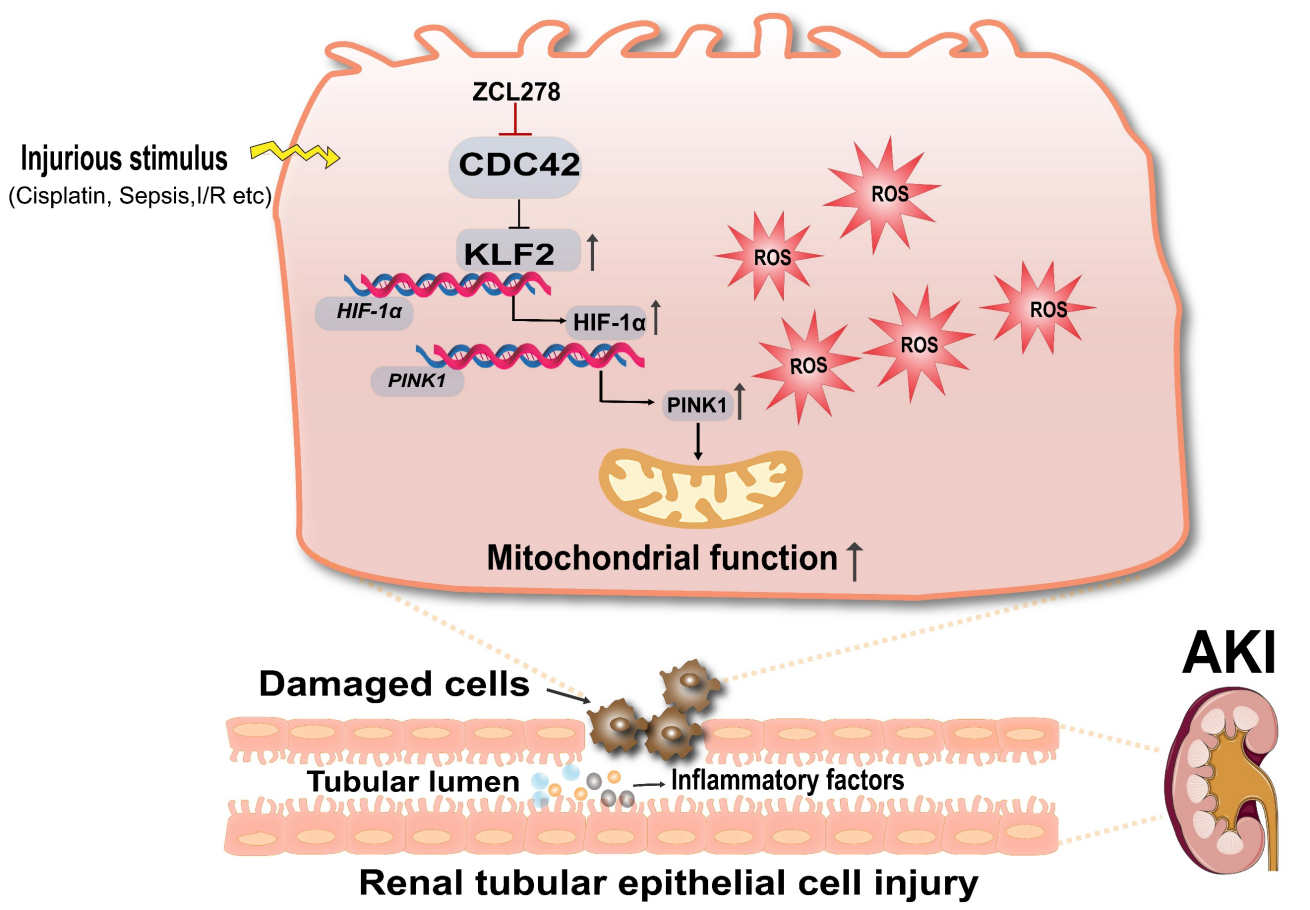
** Schematic diagram illustrating the mechanism that CDC42 inhibition protects against AKI.** Inhibition of CDC42 enhances *KLF2* promoter activity, leading to the transcriptional upregulation of *HIF-1α*, which subsequently promotes *PINK1* expression transcriptionally. Through activation of the KLF2/HIF-1α/PINK1 transcriptional cascade, CDC42 inhibition promotes mitophagy, restores mitochondrial homeostasis, and protects renal tubular epithelial cells from oxidative damage.

This study is the first to reveal that CDC42 inhibition significantly attenuated kidney injury in both CP- and I/R-induced AKI models. Mechanistically, we show that CDC42 inhibition protected RTECs *in vivo* and *in vitro* by mitigating mitochondrial dysfunction and oxidative stress via activation of the KLF2/HIF-1α/PINK1-mediated mitophagy pathway (Figure 10).

To identify candidate drivers of AKI, we first analyzed scRNA-seq data from human AKI kidney 24. KEGG enrichment revealed activation pathways related to infection and, notably, actin cytoskeleton dynamics. Given that the AKI patients developed the disease in the context of critical illnesses, severe infections, and systemic inflammation, the enrichment in actin cytoskeleton dynamics signaling pathway was particularly striking. Among the three primary regulators of actin cytoskeletal remodeling 30,* CDC42* exhibited the more pronounced dysregulation in PT cells compared to *RHOA* and *RAC1*, suggesting a central role in AKI. It is well known that CDC42 functions as a molecular switch to transduce upstream signals to downstream effectors 14-16,48, which renders it crucial for organ development 49 and disease pathogenesis 50,51, as evident by congenital defects and tumorigenesis in organ-specific knockout models 52. In oncology, CDC42 overexpression has been reported in gastric 19, colorectal 20, breast 21,22, and liver cancers 53-55, correlating with poor clinical outcomes and therapeutic resistance. Beyond cancer, CDC42 is indispensable for kidney development, as it is required for ciliogenesis in RTECs 18. Its deletion in podocyte induces congenital nephrotic syndrome 56, while in chronic kidney disease, it promotes vascular calcification and renal fibrosis 57. Despite these insights, its role in AKI pathogenesis stays unknown.

Our research showed CDC42 was upregulated in human AKI kidneys, murine models of CP- and I/R-induced AKI, and CP-treated HK-2 cells. The induction of Cdc42 was likely driven by the robust increase in inflammatory cytokines, particularly Il-6 58, which was markedly elevated (~60-120 fold) in AKI mice kidneys. Importantly, both pharmacological inhibition and genetic blockage of Cdc42 attenuated renal dysfunction (Cr, BUN, and *Kim-1*), tubular injury, and inflammatory cytokines (*Il-6, Il-1β, Tnf-α*) expression. Parallel *in vitro* studies further validated the reno-protective effects of CDC42 inhibition across experimental systems.

Mitochondrial dysfunction and the ensuing oxidative burst are well-established drivers of RTEC injury in AKI 6-9, however the potential role of CDC42 in this process remains elusive. Notably, accumulating evidences shows that actin cytoskeleton dynamics are essential for modulating mitochondrial morphology and function 59. Furthermore, recent studies indicate that glycogen synthase kinase 3β (GSK3β), a known downstream effector of CDC42 60, acts as a key regulator of mitochondrial dysfunction in kidney injury 61,62. Although the participation of GSK3β in CDC42-mediated AKI remains to be further investigated, the crosstalk between CDC42, cytoskeletal remodeling, and mitochondrial function suggests that CDC42 inhibition likely ameliorates AKI by regulating mitochondrial homeostasis. Our* in vivo* and *in vitro* studies further validated this hypothesis: CDC42 was upregulated in renal mitochondria during AKI, with its inhibition restoring mitochondrial function by rescuing ATP production, membrane potential, and ultrastructure, along with the coordinated restoration of key homeostasis regulators (PGC-1α, NRF2, MNF2, DRP1, PINK1, and PARKIN).

Transcriptomic analysis identified *KLF2* as a key downstream target of CDC42. KLF2 is a zinc finger transcription factor known to mitigate oxidative stress, inflammation, and thrombus activation 42. Previous studies have shown that KLF2 activation preserves mitochondrial function and attenuates ROS-induced damage by promoting mitophagy 40,41,63. In our study, CDC42 inhibition upregulated KLF2 expression via promoter activation, more importantly, *KLF2* deficiency abrogated the mitochondria protection afforded by CDC42 inhibition, which establishing KLF2 as an essential mediator of the CDC42-mitochondrial axis.

Further mechanistic exploration revealed that KLF2 regulates *HIF-1α* transcription. ChIP assay demonstrated *HIF-1α* is transcriptionally regulated by KLF2 at the predicted promoter binding motif, and WB analyses demonstrated that KLF2-driven HIF-1α activation in response to CDC42 inhibition. *HIF-1α* is a transcription factor with critical role in hypoxia and mitochondrial protection. Activation of HIF-1α has been found to protect against AKI 64-67 by mitigating mitochondrial dysfunction, oxidative stress, inflammation, apoptosis, and autophagy 46,68,69. In addition, multiple lines of evidence, including transcriptional database prediction, ChIP, WB, and rescue assay, validated *PINK1* was transcriptionally modulated by HIF-1α at the predicted promoter binding sites. Functionally, *PINK1* deletion exacerbated mitochondrial dysfunction and abrogated the protective effect of CDC42 inhibition, consistent with its role as a key mitophagy regulator 7,70-74. Together, these findings establish a KLF2/HIF-1α/PINK1 axis through which CDC42 inhibition promotes mitophagy and preserves mitochondrial homeostasis.

In addition to mitophagy, CDC42 inhibition dampened inflammatory responses in AKI kidneys, consistent with prior studies linking CDC42 to chronic inflammatory conditions 75-78. However, the loss of protection in *KLF2* or *PINK1* deficient models indicates that mitophagy-mediated mitochondrial preservation is the predominant protective mechanism, with anti-inflammation and other factors as the secondary outcome.

Our findings also highlight the translational potential of targeting CDC42, which is consistently upregulated in human and experimental AKI, with its inhibition conferring robust renoprotection across models. As a multifunctional signaling hub, CDC42 offers a distinct mechanistic advantage over downstream-specific strategies such as soluble epoxide hydrolase (sEH) inhibitors (anti-inflammatory) 79 or HIF-PH inhibitors (hypoxia adaptation) 80, as well as single-target approaches like PGC-1α agonists 81. By coordinately modulating key processes, including inflammation (Figure 2G, Figure 3K), oxidative stress (Figure 4J, Figure 5F), and mitochondrial function (Figure 2K-2P, Figure 2U-2W, Figure 3M-3R), CDC42 inhibition elicits synergistic effects that enable more comprehensive restoration of mitochondrial homeostasis. The selective inhibitor ZCL278, which disrupts CDC42-Intersectin interaction to suppress CDC42-mediated signaling 32, showed preventive and therapeutic efficacy without nephrotoxicity in CP- and I/R-induced AKI. These effects were further validated in RTEC-specific *Cdc42* knockdown mice, minimizing concerns regarding off-target actions and supporting CDC42 as a highly specific therapeutic target. Moreover, ZCL278 showed efficacy through multiple routes of administration, underscoring its preclinical feasibility 82. Although clinically approved CDC42-specific inhibitors are not yet available, several dual Rho GTPases inhibitors, such as R-ketorolac and R-naproxen 50,83,84, mitoxantrone 85, and MBQ-167 86 have progressed into oncology trails. These developments suggest that targeting CDC42 in AKI is both feasible and promising, warranting further exploration.

In addition, we acknowledge that the present study has certain limitations. First, our AKI models are based on rodents, and their translational relevance to human clinical settings needs further verification using more diverse clinical AKI samples. Second, the molecular mechanism underlying CDC42-mediated enhancement of *KLF2* promoter activity remains to be fully elucidated, as potential intermediate molecules may be involved.

In conclusion, our study identifies CDC42 as a pivotal regulator of AKI pathogenesis through its impact on mitochondrial function. By activating the KLF2/HIF-1α/PINK1 axis, CDC42 inhibition promotes mitophagy, restores mitochondrial homeostasis, and alleviates oxidative stress and renal injury. These findings provide mechanistic insight and translational rationale for developing CDC42-targeted and mitochondria-directed therapies for AKI.

## Supplementary Material

Supplementary methods, figures and tables.
