# Supplementary material for: Targeting CDC42 Protects Mitochondrial Function through KLF2/HIF-1α/PINK1 Signaling in Acute Kidney Injury
Source: Int J Biol Sci. 2026 Jan 1;22(3):1247–65. doi: 10.7150/ijbs.125930 (PMC12837847; doi:10.7150/ijbs.125930)
Supplement: Supplementary file 1 — Supplementary methods, figures and tables. [file ijbsv22p1247s1.pdf]

Supplementary Materials

Targeting CDC42 Protects Mitochondrial Function through KLF2/HIF-1α/PINK1 Signaling in Acute Kidney Injury

Xue Zhou [1, 2, 3], Xian Fu [2, 3], Yi-Wen Meng [2, 3], Ping Dai [2, 3], Qing Jiang [2, 3], Hou-Hua Yin [2, 3], Qing-Jin Pan [2, 3], Ai-Zhi Lin [2, 3], Kai-Di Ni [2, 3], Zi-Guo Luo [4], Ru-Yu Liang [2, 3], Yi-Yu Chen [2, 3], Hai-Xin Yuan [2, 3], Jun-Yan Liu [1, 2, 3, 5*]

[1] Medical Examination Centre of the First Affiliated Hospital and CNTTI of College of Pharmacy, Chongqing Medical University, Chongqing 400016, China

[2] Basic Medicine Research and Innovation Center for Novel Target and Therapeutic Intervention (CNTTI), Ministry of Education, Chongqing 400016, China

[3] Department of Chemical Biology, College of Pharmacy, Chongqing Medical University, Chongqing 400016, China

[4] Sci-Tech Innovation Center, Chongqing Medical University, Chongqing 400016, China

[5] Department of Anesthesia of the Second Affiliated Hospital, Chongqing Medical University, Chongqing, 400061, China

***Correspondence author**: Jun-Yan Liu, CNTTI of College of Pharmacy, Chongqing 400016, China. ORCID ID: 0000-0002-3018-0335; Tel: +86-23-6848 3587; E-mail address: jyliu@cqmu.edu.cn

Materials and Agents

Creatinine, urea, creatinine-d_3, urea-$^{15}N_2$, Cisplatin (HY-17394), ZCL278 (HY-13963), 5,6-Dichlorobenzimidazole riboside (DRB) (HY-14392) were purchased from a local distributor of MedChemExpress LLC (Shanghai, China). Primary antibodies used for Western blotting (WB) and Immunohistochemistry (IHC) included: GAPDH (1:5000, Zenbio, #R24404), HSP90 (1:5000, Proteintech, #13171-1-AP), CDC42 (1:10000, Abcam, #ab187643), KLF2 (1:500, Huabio, #ER1911-98), BCL-2 (1:1000, Zenbio, #R23309), BAX (1:8000, Proteintech, #50599-2-Ig), PARKIN (1:1000, Cell Signaling Technology, #4211S), PINK1 (1:1000, Proteintech, #23274-1-AP), NRF2 (1:1000, Cell Signaling Technology, #12721S), MFN2 (1:5000, Proteintech, #12186-1-AP), PGC-1α (1:1000, Zenbio, #381615), DRP1 (1:5000, Proteintech, #12957-1-AP), HIF-1α (1:5000, Proteintech, #20960-1-AP).

Public AKI dataset analysis

Single-cell RNA sequencing datasets were obtained from the Gene Expression Omnibus (GEO) database, including human AKI and healthy control samples from GSE210622 (1) (n = 12 samples: 8 for AKI, 4 for controls) and mouse cisplatin-induced AKI with corresponding controls from GSE197266 (2) (n = 2). Raw count matrices and metadata were processed using the Seurat R package (version 4.4.0) (3). Quality control (QC) filtered low-quality cells based on mitochondrial gene content (>5%), total unique molecular identifiers (UMIs) per cell (<500). Data were normalized using LogNormalize scaling and log transformation, with highly variable genes (HVGs; 2,000 genes) identified using the vst method. Technical variations across batches were corrected via Harmony R package (version 1.2.1) (4): principal component analysis (PCA) on HVGs was first performed, followed by Harmony integration to align cells across samples while preserving biological heterogeneity. Integrated PCA embeddings were used for dimensionality reduction via uniform manifold approximation and

projection (UMAP) with significant principal components. Cell clusters were identified through shared nearest neighbor graph-based clustering (resolution = 0.2)

Animal Protocols

Cisplatin-induced AKI mice model:

AKI was induced in 9-week-old male C57BL/6 mice by a single intraperitoneal injection of cisplatin (CP, 20 mg/kg, dissolved in saline). To investigate the preventive effect of ZCL278, mice were pre-treated with ZCL278 (30 mg/kg per day, i.p, dissolved in 10% DMSO and 90% corn oil) 24 h prior to CP administration, followed by additional doses at 24 h and 48 h after CP injection; To access the therapeutic effect, ZCL278 was administered only at 24 h and 48 h after CP injection.

To further investigate the role of the *Cdc42* gene in AKI, 9-week-old male *Cdc42*$^{f/f}$ mice and *Cdc42*$^{Cdh16\ KD}$ mice were subjected to CP-induced AKI. Control mice were treated with an equal volume of the corresponding vehicle. Blood samples (10 μL each) were collected from mice tail at 0 h, 24 h, 48 h and 72 h post-CP administration for blood urea nitrogen (BUN) and creatinine (Cr) measurement. At 72 h, mice were sacrificed following anesthesia with 2% pentobarbital (40 mg/kg), and renal tissues and orbital blood were collected for further analysis.

For the survival study, mice in the CP+ZCL group were treated with ZCL278 (i.p., 30 mg/kg) every 24 hrs post-CP administration (i.p., 20 mg/kg), while those in the CP group received an equal volume of the corresponding vehicle.

Ischemia-reperfusion (I/R)-induced AKI mice model:

The I/R-induced AKI model was established by a modified protocol based on a previous study (5). Mice were anesthetized with 2% pentobarbital (i.p., 40 mg/kg) and placed on a 37°C thermostatic pad. After hair removal and disinfection of the surgical site on the back, a dorsal incision was made, and the bilateral renal arteries were clamped with

arterial clips for 30 min. Following the ischemic period, the clips were removed, and renal blood flow restoration was confirmed before suturing the wound.

Mice in ZCL+I/R+ZCL group were administered with ZCL278 (i.p., 30 mg/kg) at 48 h and 24 h prior to I/R surgery, with an additional dose 4 h post-surgery; The sham-operated group underwent the same surgical procedure without clamping of the renal arteries. The control group did not undergo surgery. Mice were sacrificed 24 h after reperfusion, and blood and kidney tissues were collected for further analysis.

Histological Examination (H&E)

After anesthesia with 2% pentobarbital (i.p., 40 mg/kg), mice were intracardially perfused with 0.9% saline. The perfused kidney tissues were then fixed with 4% paraformaldehyde, and embedded in paraffin. Paraffin-embedded kidney sections (4 μm thick) were prepared using a paraffin microtome (Leica, Germany) and stained with H&E to examine kidney histology. Tubular damage was assessed on lumen dilatation, epithelial necrosis, cast formation, and brush border loss. The severity of tubule damage was scored on a scale of 0 to 4, according to the percentage of damaged tubules: 0, no damage; 1, <25% damage; 2, 25-50% damage; 3, 50-75% damage; 4, >75% damage. For each mouse, five random fields were analyzed using a 40x magnification objective lens. Experimenters were blinded to the experimental group during imaging and analysis.

Immunohistochemistry (IHC)

IHC was performed to evaluate the expression and distribution of Cdc42 and Klf2 in mice kidney tissues. Briefly, paraffin-embedded kidney sections (4 μm) were dewaxed in xylene and rehydrated through a gradient alcohol series. Then, antigen retrieval was performed by incubating the slides in sodium citrate antigen reparation buffer and boiling them in a microwave oven for 10 min. After cooling to room temperature, the

slides were washed three times with PBS (5 min each) and followed by incubation with 3% hydrogen peroxide for 15 min to block endogenous peroxidase activity. The slides were then blocked with 10% normal goat serum for 30 min at 37°C and incubated overnight at 4°C with primary antibody against CDC42 (Proteintech, #10155-1-AP) or KLF2 (Huabio, #ER1911-98). The next day, the sections were washed three times with PBS and then incubated with a horseradish peroxidase-conjugated anti-rabbit secondary antibody (Biosharp, #BL052A) at 37 °C for 60 min. After additional PBS washes, the sections were treated with 3,3'-diaminobenzidine (DAB) (Biosharp, #BL732A) solution for 20-35 seconds to visualize the peroxidase reaction products. Finally, the sections were counter-stained with hematoxylin and examined under a microscope. During the imaging and analysis process, the experimenters were blinded to the experimental group information.

Quantitative real-time polymerase chain reaction (q-PCR)

Total RNA was extracted from kidney cortex and HK-2 cells using TRIzol (Invitrogen, #15596018CN), followed by a cDNA synthesis using ABScript II cDNA First-Strand Synthesis Kit (ABclonal, #RK20400). Then q-PCR was performed on LightCycler480 Instrument (Roche, Switzerland) using ABScript II One Step SYBR Green RT-qPCR Kit (ABclonal, #RK20404), following the manufacturer's instructions. Fluorescence data were processed by a PCR post-data analysis software program, and *Gapdh* or *GAPDH* was used as an endogenous control for normalization. Relative gene expression levels were calculated using $2^{-\Delta\Delta CT}$ method. The primer sequences used for PCR amplification are listed in Supplementary Table S2.

Western blot analysis (WB)

Proteins from kidney tissues and HK-2 cells were extracted using RIPA lysis buffer (Beyotime, # P0013B) which contained a proteinase inhibitor (Beyotime, #P1030) and

a phosphatase inhibitor (Beyotime, #AR1183). Protein samples (20-40 μg) were first separated on 10% or 12% SDS-PAGE gels, then transferred onto PVDF membranes, and subsequently incubated with the respective primary and secondary antibodies (Biosharp, #BL052A or #BL051A). Immune complexes on the PVDF membranes were visualized using the BeyoECL Plus kit (Beyotime, #P0018M) and detected by chemiluminescence imager (CLINX, China).

Terminal deoxynucleotidyl transferase-mediated dUTP nick-end labeling (TUNEL) staining

TUNEL assay was performed to evaluate the cell apoptosis in mice kidney tissues following the manufacturer's instructions (Beyotime, #C1088). Apoptotic cells exhibiting positive staining (green) with nuclear DNA fragmentation were visualized using a fluorescence microscope (OLYMPUS, Japan), while the nuclei were counter-stained with DAPI (blue). For each mouse kidney, apoptotic cells were in five randomly selected fields. During the imaging and analysis process, the experimenters were blinded to the experimental grouping information.

Cell culture and treatments

Human renal proximal tubular cell line (HK-2 Cell) was obtained from American Type Culture Collection (ATCC, Manassas, VA, USA). Cells were cultured in DMEM/F-12 medium supplemented with 10% fetal bovine serum and 1% penicillin/streptomycin, and maintained at 37°C with 5% CO_2 in a humidified incubator. HK-2 cells were treated with 20 μm CP for 24 h to induce cell injury. To explore whether inhibition of CDC42 could produce a protective effect against CP-induced injury, HK-2 cells were pre-treated with ZCL278 (50 μm) 2 h before CP administration and then co-treated with CP for additional 24 h.

Reactive oxygen species (ROS) detection

Cells to be assayed were digested, rinsed and resuspended in a serum-free medium according to the provided instructions. Then, 10 μM DCFH-DA (diluted with serum-free medium) was added and co-incubated with cell suspension in 37°C for 30 min, with mixing every 5 min. After incubation, cells were rinsed 3 times with a serum-free medium to remove excess DCFH-DA. Finally, the cells were resuspended in a serum-free medium, and fluorescence intensity was measured immediately using a fluorescent microplate reader (Bio-Rad, USA) with an excitation wavelength of 488 nm and emission at 525 nm. The ROS detection kit was purchased from Meilunbio (#MA0219-1).

ATP measurement

The ATP content in kidney tissues and HK-2 cells was measured using the Enhanced ATP Assay Kit (Beyotime, #S0027). Briefly, kidney tissues or HK-2 cells were fully lysed by adding appropriate ATP lysis buffer and centrifuged at 4°C for 5 min at 12000 × g. Then, 100 μL of ATP assay solution was added to a 96-well opaque white plate and incubated at room temperature for 5 min to consume any background ATP before the amount of centrifugation supernatant was added. Subsequently, ATP levels were measured using the luminometer function of a multi-function microplate reader (Bio-Rad, USA). ATP levels were expressed as nmol/mg protein to eliminate differences in protein amounts.

Mitochondrial membrane potential (MMP) assay

MMP changes in HK-2 cells after CP treatment were assessed using the enhanced mitochondrial membrane potential assay kit with JC-1 (Beyotime, # C2003S) according to the manufacturer's instructions. The $\Delta\psi m$ were analyzed by Image J software, with the results were expressed as the fold change in red/green fluorescence relative to control cells.

Transmission electron microscopy (TEM)

For TEM, mice kidneys were rapidly isolated after anesthetizing with pentobarbital. The renal cortical tissue was then cut into three 1 mm pieces using a sharp blade and immersed in 4% glutaraldehyde overnight at 4°C. After that, the kidney tissues soaked in glutaraldehyde were sent to TEM center, Chongqing Medical University, for further processing, including fixation, embedding, sectioning, and staining processes by professionals. Five random micrographs were taken for each mouse using an EM-1400 PLUS transmission electron microscope, and the quantitative analysis of mitochondrial aspect ratio (major/minor axis) was performed using ImageJ software (Mean of 10 random mitochondrial per mice, n = 3 mice per group). The experimenters were blinded to the experimental grouping information during the imaging and analyses.

Cdc42-GTP pull-down assay

Cdc42 activation was examined using the Cdc42 pull-down activation assay Biochem Kit (#BK034, Cytoskeleton) following the manufacturer's instructions. Kidney tissues were lysed with lysis solution, 20 μL of protein lysates were first reserved to quantify the total Cdc42 level in each sample, while the remaining lysates were snap-frozen in liquid nitrogen. Total Cdc42 in the samples was quantified by western blot and, based on these results, the amount of total Cdc42 content in each sample was normalized. Then, an equal amount of protein lysate was incubated with PAK-PBD beads for 1 h at 4 °C, followed by centrifugation at 4000 × g for 1 min. After carefully removing the supernatant and washing the beads, GTP-Cdc42 levels were detected by western blot after suspending the beads in 20 μL of loading buffer.

Cell viability assay

Cell viability was determined by the CCK8 assay kit (Beyotime, #C0041) in accordance to the manufacturer’s instructions. Briefly, cells were seeded at a density of 4×10^3/well

in 96-well plates (100 μL medium per well) and incubated overnight. The following day, the cells were treated as indicated in "Cell culture and treatments" section and Figure legends. When cell viability assays were required, 10 μL of CCK8 solution was added and incubated at 37° C for 2 h, the absorbance at 450 nm was then detected by a microplate reader (Bio-Rad, USA). Relative viability was calculated by normalizing to untreated control cells of the same cell line as an internal reference.

Cell apoptosis assay

Cell apoptosis rate was measured using the Annexin V-FITC/PI Apoptosis Detection Kit (Solarbio, CA1020) according to the manufacturer's instructions. Treated cells were co-incubated with AnnexinV-FITC and/or propidium iodide (PI) for 15 minutes in the dark. After staining, samples were immediately assayed for apoptosis using flow cytometry and the results were processed using FlowJo v10 software.

Co-immunoprecipitation (Co-IP)

After cells were fully lysed with IP lysate (Beyotime, #P0013) containing protease inhibitors, the cell supernatant was collected by centrifugation at 14,000 × g for 10 min. Then, CDC42 antibody (Abcam, #ab187643) and the negative control IgG antibody (Beyotime, #A7058) were added and incubated overnight at 4°C. On the following day, protein A+G Agarose (Beyotime, #P2055) was added and incubated for 4 h at 4°C. After incubation, the beads were washed three times with precooled IP lysis buffer, and 1 × SDS loading buffer was then added, and the samples were boiled at 95° C for 5 min for WB assays.

Chromatin immunoprecipitation (ChIP)

ChIP assay was conducted using the Thermo Scientific™ Pierce™ Magnetic ChIP Kit (Thermo Scientific, #26157) following the manufacturer's instructions. First, cells were fixed with 1% formaldehyde for 10 min to cross-link protein and DNA, then the

crosslinking reaction was quenched with a final concentration of 1 × glycine. Next, the chromatin was digested to a length between 200-800 bp using micrococcal nuclease and sonication. The chromatin was incubated overnight at 4°C with the primary antibody and the negative control IgG antibody. The following day, magnetic beads were added and incubated at 4°C for 2 h to form an immunoprecipitation complex. Finally, the purified DNA was analyzed by q-PCR to detect DNA fragments isolated from the ChIP reaction. KLF2 antibody (Invitrogen, #PA5-40591) and HIF-1α antibody (Proteintech, #20960-1-AP) were used in ChIP assay. The primer sequences were designed precisely based on the predicted binding sites by JASPAR, specifically, the primer sequences for *HIF-1α* were：forward: 5’-CAGCAGCGCCTCCCAAAG-3’, reverse：5’-TTTAGCGGCGGAGGAAAGAG-3’; and the primer sequences for *PINK1* were ： forward: 5’-GCCTCGCTGGGGTCTTTTAA -3’, reverse ： 5’-AACCACAGGCTCACTCATGG -3’.

Dual-luciferase reporter assay

The regulatory effect of *CDC42* knockout on the *KLF2* promoter was analyzed by a dual-luciferase reporter gene assay. A full-length luciferase reporter gene for the *KLF2* (promoter 1.7-kB *KLF2*-Luc) was constructed by Tsingke Biotech Co., Ltd. (China, Beijing). The 1.7-kB *KLF2*-Luc promoter plasmid and a renilla luciferase plasmid were co-transfected into NC cells and *CDC42* KO cells. *KLF2* promoter activity in NC cells and *CDC42* KO cells was then detected using a dual luciferase assay kit (Beyotime, #RG029M), with renilla luciferase activity serving as an internal reference.

mRNA stability assay

Cells were treated with the RNA polymerase inhibitor DRB (50 μM) at a cell density of about 70%-80%. Total RNA was collected for q-PCR analysis at the following time points after DRB addition: 0, 20, 40, 60, 80, 120 and 240 min. More details of the

mRNA stability assay and the calculation of mRNA half-life are described in a previously published study (6).

References

1. Hinze C, Kocks C, Leiz J, Karaiskos N, Boltengagen A, Cao S, et al. Single-cell transcriptomics reveals common epithelial response patterns in human acute kidney injury. Genome Med. 2022 Sept 9;14(1):103.

2. Chen Z, Li Y, Yuan Y, Lai K, Ye K, Lin Y, et al. Single-cell sequencing reveals homogeneity and heterogeneity of the cytopathological mechanisms in different etiology-induced AKI. Cell Death Dis. 2023 May 11;14(5):318.

3. Butler A, Hoffman P, Smibert P, Papalexi E, Satija R. Integrating single-cell transcriptomic data across different conditions, technologies, and species. Nat Biotechnol. 2018 June;36(5):411–20.

4. Korsunsky I, Millard N, Fan J, Slowikowski K, Zhang F, Wei K, et al. Fast, sensitive and accurate integration of single-cell data with Harmony. Nat Methods. 2019 Dec;16(12):1289–96.

5. Deng BQ, Luo Y, Kang X, Li CB, Morisseau C, Yang J, et al. Epoxide metabolites of arachidonate and docosahexaenoate function conversely in acute kidney injury involved in GSK3β signaling. Proc Natl Acad Sci. 2017 Nov 21;114(47):12608–13.

6. Ratnadiwakara M, Änkö ML. mRNA Stability Assay Using transcription inhibition by Actinomycin D in Mouse Pluripotent Stem Cells. Bio-Protoc. 2018 Nov 5;8(21):e3072.

Supplementary Table S1 Primer sequences for mice gene identification

Primer sequences for identification of Cdh16-Cre mice

Primer	Sequence (5’→ 3’)
P1	GCAGATCTGGCTCTCCAAAG
P2	AGGCAAATTTTGGTGTACGG
P3	CAAATGTTGCTTGTCTGGTG
P4	GTCAGTCGAGTGCACAGTTT

Primer sequences for identification of *Cdc42*$^{flox/flox}$ mice

Primer	Sequence (5’→ 3’)
P1	AAAGTTTTTAGACTTGAAGGTGGTT
P2	GGCAGCAAAGGAAAAGTGCTA

Supplementary Table S2 Primer sequences for q-PCR

Genes	Forward primer (5’ → 3’)	Reverse primer (5’ → 3’)
human		
GAPDH	**F:** GGAGCGAGATCCCTCCAAAAT	**R:** GGCTGTTGTCATACTTCTCATGG
CDC42	**F:** CCATCGGAATATGTACCGACTG	**R:** CTCAGCGGTCGTAATCTGTCA
KLF2	**F:** ACTCACACCTGCAGCTACGC	**R:** AGTGGTAGGGCTTCTCACCTGT
KIM-1	**F:** TGGCAGATTCTGTAGCTGGTT	**R:** AGAGAACATGAGCCTCTATTCCA
TNF-α	**F:** GAGGCCAAGCCCTGGTATG	**R:** CGGGCCGATTGATCTCAGC
IL-1β	**F:** ATGATGGCTTATTACAGTGGCAA	**R:** GTCGGAGATTCGTAGCTGGA
IL-6	**F:** ACTCACCTCTTCAGAACGAATTG	**R:** CCATCTTTGGAAGGTTCAGGTTG
mice		
Gapdh	**F:** AATGGATTTGGACGCATTGGT	**R:** TTTGCACTGGTACGTGTTGAT
Cdc42	**F:** CCCATCGGAATATGTACCAACTG	**R:** CGGTCGTAGTCTGTCATAATCCT
Klf2	**F:** GAGCCTATCTTGCCGTCCTTT	**R:** CACGTTGTTTAGGTCCTCATCC
Kim-1	**F:** AGCAGTCGGTACAACTTAAAGG	**R:** ACTCGACAACAATACAGACCAC
Tnf-α	**F:** CCAGACCCTCACACTCAGAT	**R:** AAC ACCCAT TCCCTTCACAG
Il-1β	**F:** ATGGCAACTGTTCCTGAACTCAACT	**R:** CAGGACAGGTATAGATTCTTTCCTTT
Il-6	**F:** GGCGGATCGGATGTTGTGAT	**R:** GGACCCCAGACAATCGGTTG

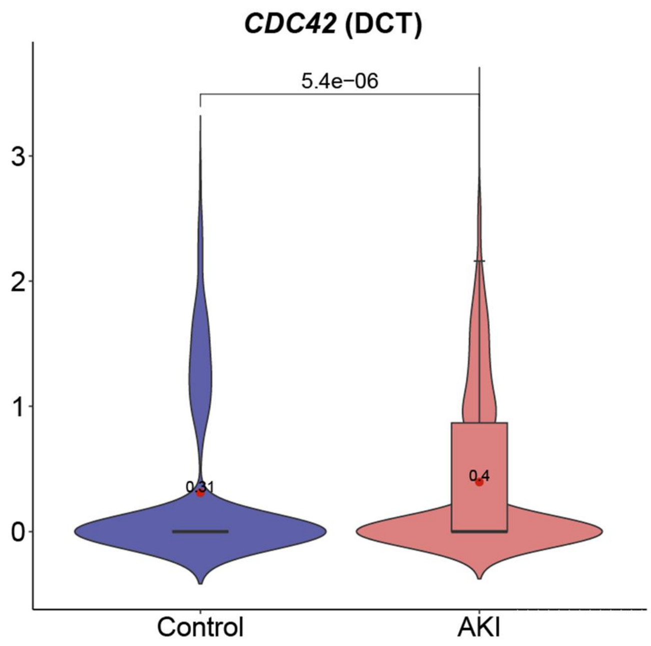

Supplementary Figure S1. *CDC42* expression was significantly upregulated in DCT cells of human AKI patients.

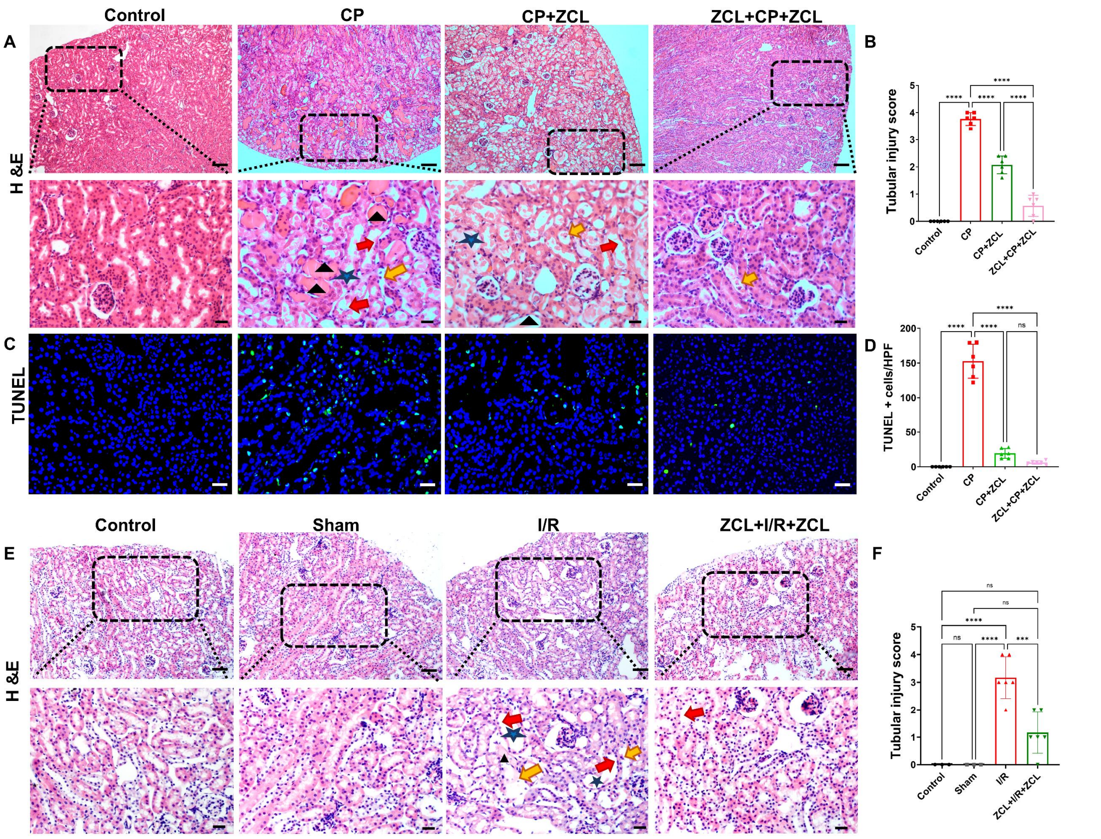

Supplementary Figure S2. Inhibition of Cdc42 alleviated kidney injury in CP- and I/R induced AKI mice. (A) Representative images of H&E staining (scale bar, 100 μm) and the corresponding magnified images (scale bar, 20 μm) in mouse kidney sections showed ZCL278 attenuated kidney injury caused by CP challenge (black arrows: cast formation, red arrows: loss of brush border, yellow arrows: necrosis of epithelial cell, blue pentagram: lumen dilatation; n = 6); **(B)** ZCL278 decreased renal tubular injury scores in CP-induced AKI mice; **(C)** Representative images of TUNEL staining indicated ZCL278 treatment decreased mice renal apoptotic cells caused by CP challenge (Green and blue staining indicated TUNEL-positive cells and nuclei, respectively; n = 6, scale bar, 20 μm); **(D)** Quantitative results of **(C)**; **(E-F)** Representative images of H&E staining (scale bar, 50 μm) and the corresponding magnified images (scale bar, 20 μm) in mouse kidney sections showed ZCL278 attenuated kidney injury in I/R-induced AKI mice (n = 3 in control and sham group, n=6 in I/R and ZCL+I/R+ZCL group). Data were presented as means ± SD. * $0.01 < p \leq 0.05$, ** $0.001 < p \leq 0.01$, *** $0.0001 < p \leq 0.001$, **** $p \leq 0.0001$.

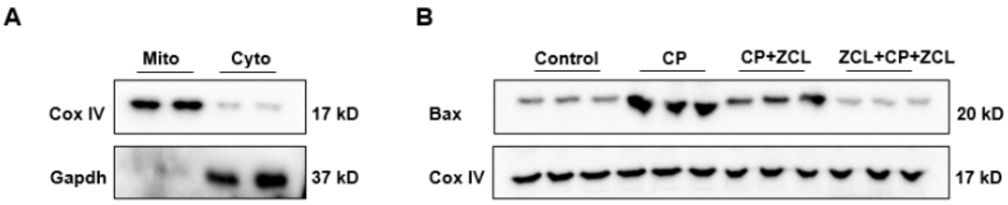

Supplementary Figure S3. Inhibition of Cdc42 decreased the increase in Bax protein expression in the renal mitochondria of CP-AKI mice. (A) Mitochondria were isolated from mouse kidneys (n = 3); **(B)** ZCL278 treatment decreased the increase in Bax protein expression in the renal mitochondria of CP-AKI mice (n = 3).

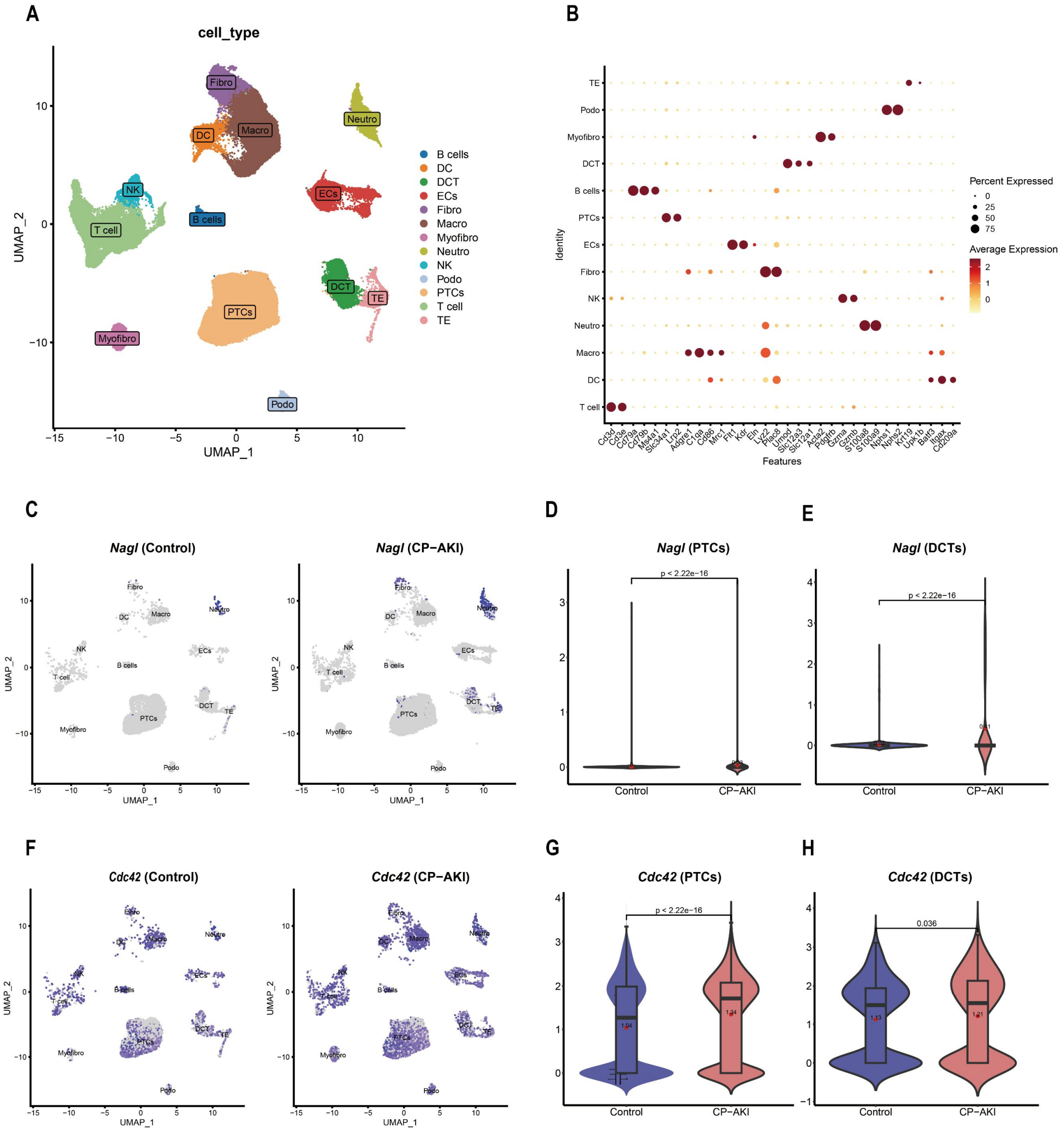

Supplementary Figure S4. ScRNA-seq data of control mice and CP-AKI mice revealed that *Nagl* and *Cdc42* expression were upregulated in PT and DCT cells. **(A)** UMAP of 17907 cells from control group and CP-AKI group, data were originated from the scRNA-seq dataset reported by Chen et al (2) (Myofibro, myofibroblasts; Macro, macrophage; Podo, podocytes; NK, natural killer cells; DC, dendritic cells; PTCs, proximal tubular cells; ECs, endothelial cells; Neutro, neutrophil; DCTs, distal convoluted tubules; TE, transitional epithelia); **(B)** Dot plot showing the representative maker genes of each cell cluster; **(C)** UMAP showing *Nagl* expression pattern in control mouse kidneys (left) and CP-AKI mouse kidneys (right); Violin plot demonstrated that *Nagl* expression was significantly upregulated in PTCs **(D)** and DCTs **(E)** of the CP-AKI group. These data indicate CP exposure caused greater damage to DCT cells than PT cells; **(F)** UMAP showing *Cdc42* expression pattern in control mouse kidneys (left) and CP-AKI mouse kidneys (right); Violin plot demonstrated that *Cdc42* expression was significantly upregulated in PTCs **(G)** and DCTs **(H)** of the CP-AKI group. These data indicate CP exposure caused similar upregulation of *Cdc42* in DCT cells and PT cells.

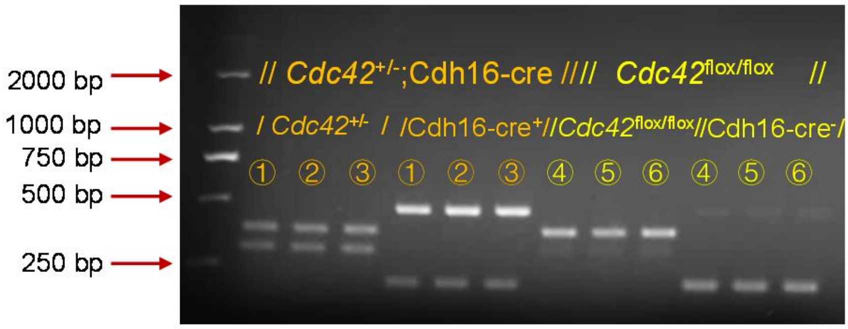

Supplementary Figure S5. The genotypes of *Cdc42*$^{+/-}$; Cdh16-Cre mice and *Cdc42*$^{flox/flox}$ mice were identified by PCR. The genotypes of mice numbered ①, ②, ③ were identified as *Cdc42*$^{+/-}$; Cdh16-cre, and mice numbered ④, ⑤, ⑥ were identified as *Cdc42*$^{flox/flox}$.

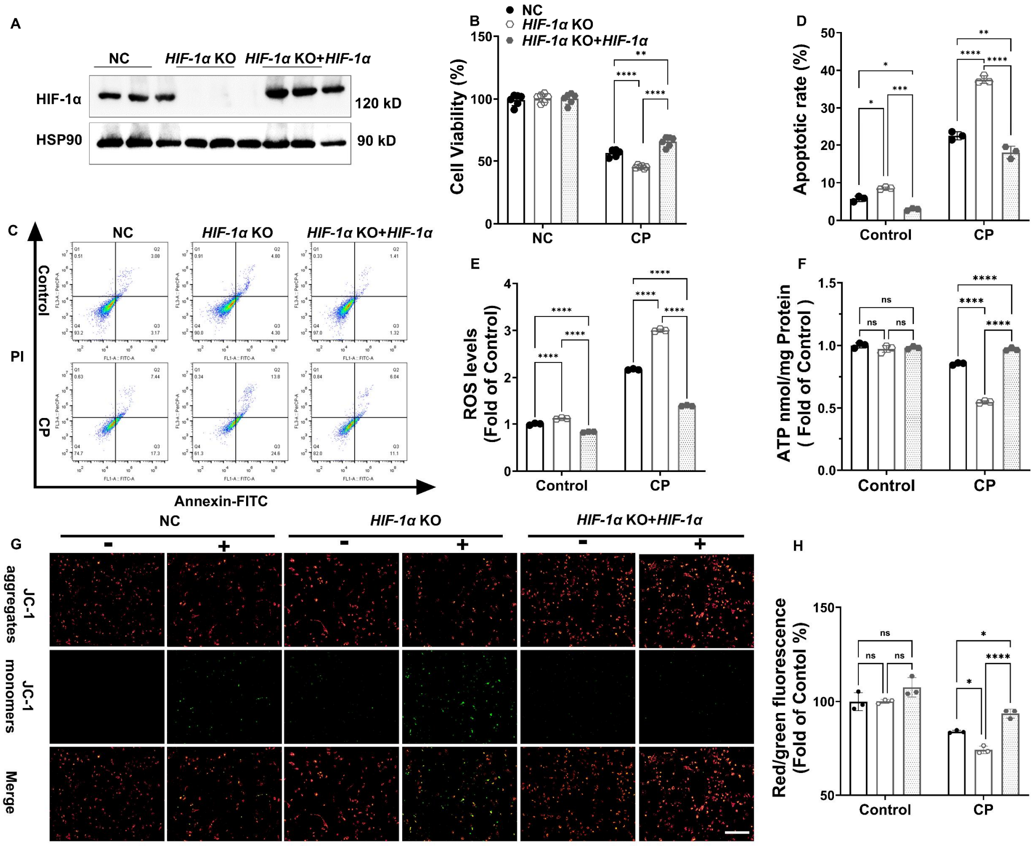

Supplementary Figure S6. Re-expression of *HIF-1α* rescued cell damage and mitochondrial dysfunction caused by CP exposure. **(A)** *HIF-1α* was re-expressed in *HIF-1α* KO cells; **(B)** CP-induced decrease in cell viability in *HIF-1α* KO cells was restored by re-expression of *HIF-1α* KO (n = 6); **(C-D)** *HIF-1α* re-expression significantly reduced cell apoptosis rate in *HIF-1α* KO cells (n = 3); **(E)** Re-expression of *HIF-1α* decreased cellular ROS concentration (n = 3); **(F-H)** Impaired mitochondrial function in *HIF-1α* KO cells was reversed in *HIF-1α* KO + *HIF-1α* cells (n = 3). Data were presented as means ± SD. * $0.01 < p \leq 0.05$, ** $0.001 < p \leq 0.01$, *** $0.0001 < p \leq 0.001$, **** $p \leq 0.0001$.

A *PINK1*

Display 10 profiles

Matrix ID	Name	Score	Relative score
MA1106.1	MA1106.1.HIF1A	11.35618	0.9595118265456497
MA1106.1	MA1106.1.HIF1A	7.438316	0.8672622881880742
MA1106.1	MA1106.1.HIF1A	7.171165	0.8609719873452479
MA1106.1	MA1106.1.HIF1A	6.376037	0.842250007006588
MA1106.1	MA1106.1.HIF1A	6.296889	0.8403863913100182

B *PARKIN*

Display 10 profiles

Matrix ID	Name	Score	Relative score
MA1106.1	MA1106.1.HIF1A	7.8345604	0.8765922117335178
MA1106.1	MA1106.1.HIF1A	5.9282236	0.8317058466559162

C *NRF2*

Display 10 profiles

Matrix ID	Name	Score	Relative score
MA1106.1	MA1106.1.HIF1A	9.960091	0.9266396782933561
MA1106.1	MA1106.1.HIF1A	8.547723	0.8933842440250763
MA1106.1	MA1106.1.HIF1A	7.674064	0.8728131876638376
MA1106.1	MA1106.1.HIF1A	7.167511	0.8608859506488831
MA1106.1	MA1106.1.HIF1A	5.6976514	0.8262768222080843

D *PGC-1α*

Display 10 profiles

Matrix ID	Name	Score	Relative score
MA1106.1	MA1106.1.HIF1A	8.558895	0.8936473054626111
MA1106.1	MA1106.1.HIF1A	7.7372575	0.8743011296216148
MA1106.1	MA1106.1.HIF1A	7.2596793	0.8630561345294854
MA1106.1	MA1106.1.HIF1A	7.0967126	0.8592189405362941
MA1106.1	MA1106.1.HIF1A	7.0037036	0.8570289624894787

E *MFN2*

Display 10 profiles

Matrix ID	Name	Score	Relative score
MA1106.1	MA1106.1.HIF1A	7.96717	0.8797146151437296
MA1106.1	MA1106.1.HIF1A	7.227545	0.8622994988770356
MA1106.1	MA1106.1.HIF1A	6.336034	0.8413080943655681
MA1106.1	MA1106.1.HIF1A	5.8820877	0.830619536526713
MA1106.1	MA1106.1.HIF1A	5.67556	0.8257566611607287

F *DRP1*

Display 10 profiles

Matrix ID	Name	Score	Relative score
MA1106.1	MA1106.1.HIF1A	5.3301373	0.817623380853607
MA1106.1	MA1106.1.HIF1A	5.264571	0.8160795706195527

Supplementary Figure S7. Investigation in the JASPER database indicated that *PINK1* is most likely to be transcriptionally regulated by HIF-1α. Predicted scores for *PINK1* **(A)**, *PARKIN* **(B)**, *NRF2* **(C)**, *PGC-1α* **(D)**, *MFN2* **(E)** and *DRP1* **(F)** to be potentially transcriptionally regulated by HIF-1α using the JASPER database.
